# Dose-Finding Study of Omeprazole on Gastric pH in Neonates with Gastro-Esophageal Acid Reflux Using a Bayesian Sequential Approach

**DOI:** 10.1371/journal.pone.0166207

**Published:** 2016-12-21

**Authors:** Florentia Kaguelidou, Corinne Alberti, Valerie Biran, Olivier Bourdon, Caroline Farnoux, Sarah Zohar, Evelyne Jacqz-Aigrain

**Affiliations:** 1 Assistance Publique - Hôpitaux de Paris, Department of Pediatric Pharmacology and Pharmacogenetics and INSERM CIC1426, Hôpital Robert Debré, Paris, France; 2 Univ Paris 7-Diderot, Sorbonne Paris Cité, ED 563-EA7323, Paris, France; 3 Assistance Publique - Hôpitaux de Paris, Department of Clinical Epidemiology and INSERM CIC1426 Hôpital Robert Debré, Paris, France; 4 Univ Paris 7-Diderot, Sorbonne Paris Cité, France UMR-S 1123 and ECEVE, Paris, France; 5 Assistance Publique - Hôpitaux de Paris, Department of Neonatology Hôpital Robert Debré, Paris, France; 6 Univ Paris 7-Diderot, Sorbonne Paris Cité, France UMR-S 1123, Paris, France; 7 Assistance Publique - Hôpitaux de Paris, Department of Pharmacy Hôpital Robert Debré, Paris, France; 8 Univ Paris 5-Descartes and Univ Paris 13 Sorbonne Paris Cité Laboratoire Educations et Pratiques de Santé, EA 3412, Paris, France; 9 Univ Paris 6 - Pierre et Marie Curie, Paris, France; 10 INSERM, Paris France U1138 Centre de Recherche des Cordeliers, Paris, France; University of Nottingham, UNITED KINGDOM

## Abstract

**Objective:**

Proton pump inhibitors are frequently administered on clinical symptoms in neonates but benefit remains controversial. Clinical trials validating omeprazole dosage in neonates are limited. The objective of this trial was to determine the minimum effective dose (MED) of omeprazole to treat pathological acid reflux in neonates using reflux index as surrogate marker.

**Design:**

Double blind dose-finding trial with continual reassessment method of individual dose administration using a Bayesian approach, aiming to select drug dose as close as possible to the predefined target level of efficacy (with a credibility interval of 95%).

**Setting:**

Neonatal Intensive Care unit of the Robert Debré University Hospital in Paris, France.

**Patients:**

Neonates with a postmenstrual age ≥ 35 weeks and a pathologic 24-hour intra-esophageal pH monitoring defined by a reflux index ≥ 5% over 24 hours were considered for participation. Recruitment was stratified to 3 groups according to gestational age at birth.

**Intervention:**

Five preselected doses of oral omeprazole from 1 to 3 mg/kg/day.

**Main outcome measures:**

Primary outcome, measured at 35 weeks postmenstrual age or more, was a reflux index <5% during the 24-h pH monitoring registered 72±24 hours after omeprazole initiation.

**Results:**

Fifty-four neonates with a reflux index ranging from 5.06 to 27.7% were included. Median age was 37.5 days and median postmenstrual age was 36 weeks. In neonates born at less than 32 weeks of GA (n = 30), the MED was 2.5mg/kg/day with an estimated mean *posterior* probability of success of 97.7% (95% credibility interval: 90.3–99.7%). The MED was 1mg/kg/day for neonates born at more than 32 GA (n = 24).

**Conclusions:**

Omeprazole is extensively prescribed on clinical symptoms but efficacy is not demonstrated while safety concerns do exist. When treatment is required, the daily dose needs to be validated in preterm and term neonates. Optimal doses of omeprazole to increase gastric pH and decrease reflux index below 5% over 24 hours, determined using an adaptive Bayesian design differ among neonates. Both gestational and postnatal ages account for these differences but their differential impact on omeprazole doses remains to be determined.

## Introduction

All newborns, even very premature, are able to maintain an acid gastric pH from the first day of life, although the overall level of acid secretion may be inferior in preterm that in term neonates or infants [1,2]. In neonates, acid reflux seems a major factor contributing to gastroesophageal reflux disease (GERD). Although clinical efficacy of the different proton pump inhibitors (PPI) has not been demonstrated and benefit remains controversial, omeprazole is extensively prescribed for the treatment of this disorder [3, 4].

Dose requirements in neonates may differ between preterm and term neonates for most drugs and should consider all developmental aspects of drug disposition and effects, including developmental pharmacokinetics and ontogeny of proton pump H^+^/K^+^-adenosine triphosphatase (ATPase) [5–7].

As no trial has defined the appropriate omeprazole dose required in neonates to treat acid reflux disease, [8, 9], the aim of this trial was to determine the minimum effective dose (MED) to treat pathological GERD, using reflux index as surrogate marker and the continual reassessment method in a Bayesian framework. We also determined its short-term safety.

## Methods

### The continual reassessment method

The continual reassessment method based on Bayesian inference aims to determine the adequate dose to obtain a level (probability) of efficacy (success) as close as possible to the predetermined target level of efficacy in the population studied. Initially, (prior to patients’ inclusion), a mean *prior* probability of success is selected for different drug doses and then, after analysis of individual response, the dose to be allocating to the next patient (or cohort of patients) is based on the *posterior* probabilily of success. The mean *posterior* probability of success of each dose level is re-estimated after each cohort of three patients on the basis of observed response. The dose allocated to each new cohort of patients is the one with the updated response probability closest to the predefined target.

In the present study, five dosages of omeprazole were selected from 1 to 3 mg/kg/day and each was associated with a mean *prior* probability of success according to the clinical experience of three senior neonatologists and pharmacologist (CF, VB, EJA) and to literature data. The following mean *prior* probabilities were given: dose of 1 mg/kg/day: 50%; 1.5 mg/kg/day: 70%; 2 mg/kg/day: 85%; 2.5 mg/kg/day: 95%; and 3 mg/kg/day: 100%. The target probability of success was defined to be 95%. To assess the influence of gestational age (GA) on omeprazole’s efficacy, patients were stratified in 3 groups (less than 32 weeks, 32 to 35 weeks and more than 35 weeks’ GA) and the maximum number of neonates to be studied was 30 neonates per group [10–13]. For each group, starting dose was randomly selected by the study biostatistician. Then, in each group, three consecutive patients received the same dose, as determined by the statistician on the basis of preceding cohort results.

### Study protocol

The trial was conducted in the neonatal intensive care unit of Robert Debré University Hospital, Paris, France. Study was approved by the Ethics Committee (Comité de Protection des Personnes), Hôpital Saint Louis, Paris, France (n°: 06-12-07, January 15^th^ 2007) and was registered in the EUDRACT database (n°: 2006-005335-16). All neonates were included after parental signed consent.

1. Inclusion criteria included postmenstrual age ≥ 35 weeks with a pathologic 24-hour intra-esophageal pH monitoring. The latter was defined by a reflux index (RI, percentage of the entire record with intra-esophageal pH is <4) superior or equal to 5%. In our unit, 24h-pH monitoring is performed only in neonates with a postmenstrual age ≥ 35 weeks because of tube feeding and it is either prescribed systematically or following medical decision in the presence of clinical symptoms of GERD (vomiting oxygen, desaturation and bradycardia.,).
2. Exclusion criteria included treatment with a PPI within a week of inclusion, acute gastro-intestinal disease, metabolic and biological disorders and co-administration of atazanavir or ritonavir.
3. For pH monitoring, a single-use pediatric pH catheter with a pH-sensitive antimony electrode (VersaFlex^®^, Sierra Scientific Instruments) was placed in the lower esophagus through nasal insertion after a 3-hour fasting period. Positioning of the catheter was calculated using a body height formula (Strobel formula) or checked by chest X-ray. Recordings were performed with an external device (Ambulatory pH recorder, Orion II, Medical Measurement Systems) and pH curves were analyzed using appropriate software (GastroTrac^®^, Alpine Biomed Corp. and Medical Measurement Systems). In addition, oro-pharyngeal pH monitoring was performed using litmus paper (VWR International, France)
4. Following inclusion, omeprazole was administered orally, once a day in the morning for 5 days. Five omeprazole capsules made of granules, were prepared by the hospital pharmacist. Capsules were opened and the content was mixed in formula milk. Study personnel involved in every day medical care and parents were blinded to the dose allocated.

Patients’ participation ended after completion of a second 24-h intra-esophageal pH monitoring at 72±24 hours after treatment initiation. Non-pharmacological therapies for the management of GERD, flat prone positioning and use of formula thickening agents, were allowed. However, use of pharmacological agents other than omeprazole was prohibited.

The primary outcome was a normalized 24-h intra-esophageal pH monitoring after 72±24 hours of omeprazole treatment, defined by a reflux index of less than 5%. Secondary outcomes included: mean number of reflux episodes per hour, duration of longest reflux episode, changes in salivary pH monitoring before and after treatment and evaluation of short-term safety on daily clinical examination and routine laboratory measurements.

### Statistical analysis

The two-stage sequential Bayesian analysis has been previously described in detail [14]. Briefly, the first-stage analysis, based on the continual reassessment method (CRM), aims to estimate the MED. The CRM continued until one of the following discontinuation criteria was met: 1) the planned number of 30 patients per subgroup is reached; 2) the estimated response probability is too low for all dose levels; 3) suitable estimation of the MED is obtained based on the predictive gains of further patients inclusions on the response probability. The second stage analysis ensures that the trial provides reliable estimations in case of early termination. Thus, inclusions are pursued at the MED level to obtain reliable estimates of its probability of success (95% credibility interval, measuring the degree of certainty of the MED). Again, the analysis may terminate earlier according to predefined rules. Early termination of the study is to be validated by the investigator, the sponsor and the independent monitoring board of the study.

In the present work, because of difficulties to recruit neonates born at more than 35 weeks GA, analysis was conducted by combining data with those of neonates born between 32 and 35 weeks GA. This was performed through a meta-analysis of the dose and outcomes observed in the two groups [15].

Intra-group comparisons before and after administration of omeprazole were performed using a Wilcoxon signed rank test or conditional logistic regression models. Descriptive statistical analyses were performed with the SAS 9.2 (SAS Inc, Cary, NC, USA) software package for PC and Bayesian statistical analysis with a program written in C language [16].

## Results

### Participants

**Fig 1:**
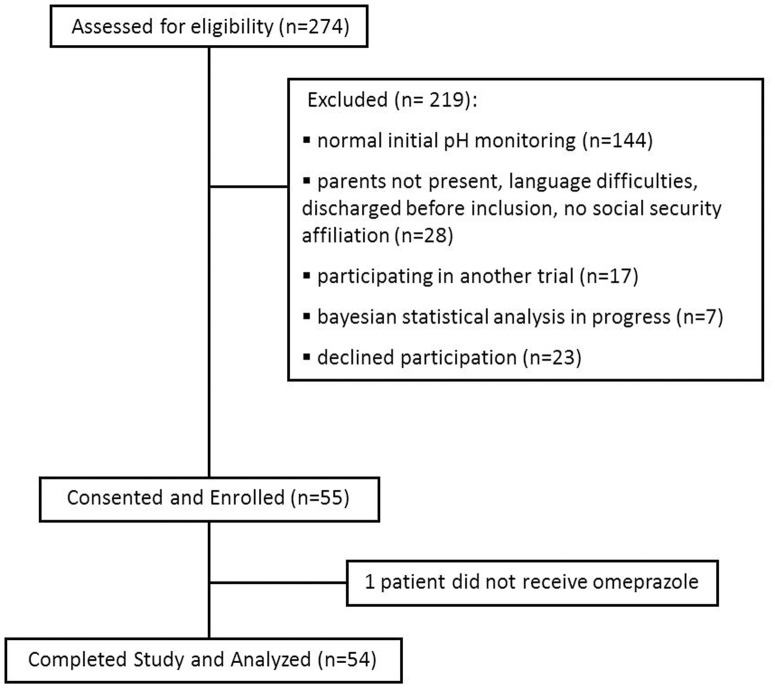
Flow chart of patients.

Overall, 274 neonates were screened for eligibility, 55 met the entry criteria and 54 completed the study and were analyzed as presented Fig 1.

Median postnatal age at inclusion was 37.5 days and median post-menstrual age was 36 weeks. The majority presented with vomiting (30/54; 56%) or other clinical symptoms suggestive of GERD, mainly oxygen desaturation and bradycardia. Forty-nine neonates were under concomitant therapy that remained stable throughout the study: vitamins (n = 46; vitamin D and K, iron and folic acid), antibiotics (n = 1; metronidazole), antifungal medication (n = 2; econazole and mycostatin) and metoclopramide (n = 5). Postnatal age was inversely correlated to GA (Spearmans’ *r* = -0.86, *p* = <0.0001) and neonates born at less than 32 weeks were older at study inclusion. Characteristics of participating neonates are shown in Table 1.

**Table 1:** Characteristics of neonates included in the study.

Patient characteristics (n = 54)		All	GA < 32 weeks	GA ≥ 32 weeks
**N**		54	30	24
**Sex ratio (male/female)**		25/29 (46%/54%)	10/20 (33%/67%)	15/9 (63%/37%)
**Gestational age (weeks)**		31 (24–41)	29 (24–31)	33 (32–41)
Postnatal age (days)**		37.5 (6–85)	48.5 (32–85)	25.5 (6–53)
Postmenstrual age (weeks)**		36 (34–43)	35 (34–39)	346 (34–43)
**Weight (g)**		2112.5 (780–3800)	2035 (1840–2330)	2140 (1892–2670)
**GERD symptoms**	**Isolated vomiting**	16 (30%)	5 (17%)	11 (46%)
	**complicated vomiting**°	14 (26%)	10 (33%)	4 (17%)
	**other clinical symptoms of GERD**°°	17 (31%)	9 (30%)	8 (33%)
	**routine pH monitoring**	7 (13%)	6 (20%)	1 (4%)
**Concomitant drug therapy**	**yes**		27 (90%)	22 (92%)
	**no**		3 (10%)	2 (8%)
**Duration of hospitalization (days)**			54 (42–75)	27.5 (22–33)

Values are expressed in number (percentage) or median (range)

° association of vomiting with apnea and/or bradycardia

°° oxygen desaturation, bradycardia or both, apnea, polypnea, stomach blood residues

** at treatment initiation

### Efficacy assessment and dose finding

At the initial 24-h pH monitoring, RI ranged from 5.06% to 27.7%. Twenty five neonates (46%) had a RI ≥ 10%. There was no difference between the two GA groups in severity of the initial acid reflux disease as assessed by the RI, the time of pH<4, the mean number of reflux episodes per hour and the duration of the longest reflux episode (data not shown).

A significant improvement was observed in all parameters of the esophageal pH monitoring, from baseline to 72 ± 12 hours after omeprazole initiation (Table 2).

**Table 2:** Efficacy parameters at baseline and 72 (±12) hours after omeprazole initiation.

	Variables	Baseline	72 ± 12 hours after omeprazole initiation	p- values
**Intra-esophageal pH monitoring**	**Reflux Index (%)**	9.7 (7.5–16.7)	0.2 (0–1.5)	***< 0*.*0001***
	**Time with pH <4 (min)**	120.5 (81–198)	2 (0–20)	***< 0*.*0001***
	**Mean number of reflux episodes per hour**	2.9 (2–4.7)	0.3 (0.1–1)	***0*.*0002***
	**Duration of the longest reflux episode (min)**	42 (22–76)	2 (0.2–11)	***0*.*0009***
Oro-pharyngeal pH monitoring*	**Value of oro-pharyngeal pH**	5.5 (5.3–6.1)	5.5 (5–6)	***0*.*04***

*missing values: n = 6

Values are expressed in median (first—third quartile, Q_1_—Q_3_)

**Fig 2:**
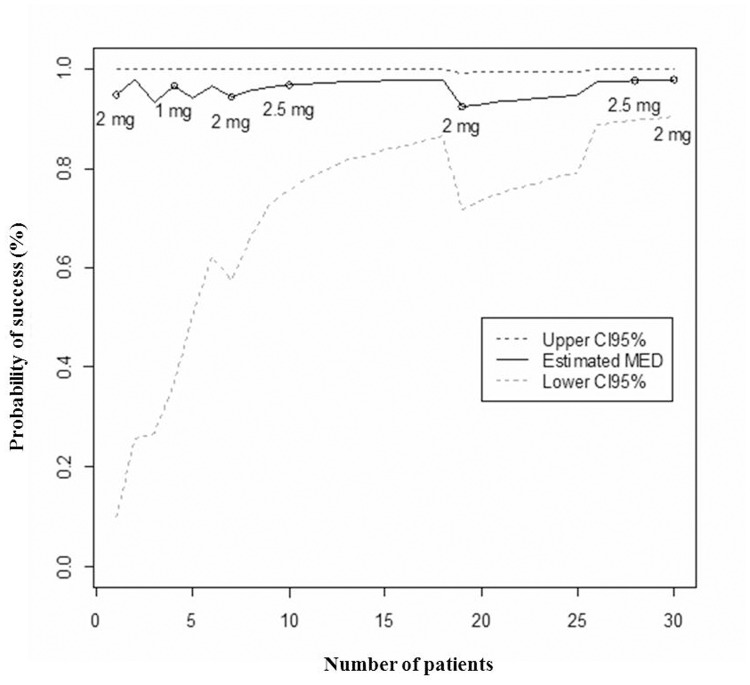
Mean posterior probability of success related to the minimum effective dose and its 95% credibility interval according to patients’ inclusions for the group of neonates born below 32 weeks of gestational age. CI95%: 95% credibililty interval. MED: Minimum Effective Dose.

Among neonates with GA<32 weeks (n = 30), 3 (10%) received a dose of 1mg/kg/day, 16 (53%) a dose of 2mg/kg/day and 11 (37%) neonates a dose of 2.5 mg/kg/day. All but three neonates (one in the 1mg/kg/day and two in the 2 mg/kg/day group) responded to omeprazole treatment (RI<5% after 72±24 hours of omeprazole treatment) (Table 3). The MED was found to be 2.5 mg/kg/day with an estimated mean *posterior* probability of success of 97.7% (95% credibility interval: 90.3%—99.7%) (Fig 2).

**Table 3:** Sequential estimation of posterior probabilities of success after the inclusion of each group of three patients in the group of neonates born at less than 32 weeks of gestational age.

			Dose (mg/kg)				
			1	1.5	2	2.5	3
			*Mean prior probabilities of success*				
			0.5	0.7	0.85	0.95	0.99
Number of cohort (3 neonates)	Dose (mg/kg daily)	Success	*Mean posterior probabilities of success*				
1	2	3/3	**0.931**	0.985	0.997	1	1
2	1	2/3	0.73	0.893	**0.965**	0.993	1
3	2	2/3	0.54	0.741	0.879	**0.963**	1
4	2.5	3/3	0.568	0.769	0.897	**0.97**	1
5	2.5	3/3	0.589	0.787	**0.909**	0.975	1
6	2.5	3/3	0.606	0.802	**0.918**	0.978	1
7	2	3/3	0.638	0.828	**0.933**	0.983	1
8	2	3/3	0.633	0.848	**0.943**	0.986	1
9	2	2/3	0.586	0.784	0.907	**0.974**	1
10*	2.5	2/2	0.591	0.789	0.91	**0.975**	1
11*	2	1/1	0.598	0.795	0.914	**0.977**	1

Bold characters indicate the estimations of the minimum efficient dose after the inclusion of each cohort.

Mean posterior probabilities of success for each dose are highlighted in grey.

* The cohort n° = 10 included only 2 neonates that received a daily dose of 2.5 mg/kg and the cohort n° = 11 included only 1 infant that received a daily dose of 2mg/kg because of an error of dose allocation

Individual data and sequential estimations of the mean *posterior* probabilities of success are presented in Table 3. As a total of 30 neonates were included in this group, no second stage analysis was required.

Among neonates with GA >32 weeks (n = 24), 18 (75%) were treated with 1 mg/kg and 6 (25%) with 2 mg/kg daily. All neonates but one (in the 1 mg/kg group) responded to the omeprazole treatment. In this group, a second-stage analysis was required and the pooled MED was found to be 1mg/kg/day. Sequential estimations of mean *posterior* probabilities of success after each cohort are detailed in S1 Table and S1 Fig for neonates born between 32 and 35 weeks of gestational age, in S2 Table in the group of neonates born after 35 weeks of gestational age. S3 Table provides the pooled estimation of the minimum effective dose in the group of neonates born after 32 weeks of gestational age.

### Safety assessment

Omeprazole was well tolerated clinically and with respect to laboratory tests. Clinical adverse events were reported in 9 neonates and included persistence of vomiting (n = 5), apnea (n = 3), tachycardia (n = 1), bradycardia with oxygen desaturation (n = 4), anal fissure with blood in the stool (n = 1). None was attributed to omeprazole nor required treatment discontinuation. All presented a RI <5% after 72±24 hours of omeprazole treatment except two neonates (one in each GA group) who presented a RI of 9.3% and 5% respectively, the first one with bradycardia and oxygen desaturation and the second one with persisting vomiting,.

## Discussion

This is the first dose-finding trial for omeprazole in neonates using an adaptive Bayesian statistical method. The results show that the minimum effective dose depends on gestational age at birth and postnatal age. Optimal dose was higher in older neonates (and born very prematurely) than in younger neonates (and born less prematurely).

According to our findings, the MEDs were different in the two studied “age groups”, and this may depend on different factors [5–7]. 1) Gastric acid secretion does not reach adult levels before six months of age thus suggesting a lower per kilogram efficient dose of omeprazole in neonates than in children or adults [17]. However, this contrasts with the daily doses up to 2 to 3 mg/kg currently used by neonatologists. 2) Age-related differences in omeprazole disposition were already reported [18, 19], yet the impact of gestational and postnatal ages were not evaluated [17], but both are known to influence pre- and postnatal physiological maturation of renal function [20, 21]. Immaturity of the proton pumps is unlikely as even in extremely premature neonates, gastric pH is below 4 from birth and proton pumps functional as early as 13 weeks of gestation [22, 23]; 3) Disease and medical care. Premature neonates receive frequent feeds and the buffering effect of milk may affect conversion of the pro-drug to its active form that binds to the proton pump [24, 25]. Frequency of meals, but also medical conditions and medications may explain differences in omeprazole optimal dose between the two groups, in agreement with previous report[26].

There are potential risks related to the use of inhibitors of gastric acidity. Increase of neonatal intestinal and pulmonary infections and occurrence of severe hypomagnesaemia have been described [27–31]. Deleterious drug effects emphasize the importance of assessing the minimum effective dose of PPIs in neonates but pharmacokinetic/pharmacodynamic studies are often difficult to conduct in neonatology. Clinical trials conducted according to innovative Bayesian inference methodology should be further developed, because they are more flexible [32] and suited to clinical situations where clinicians have acquired a strong opinion on drug efficacy through personal experience.

GERD may cause troublesome symptoms and/or complications, such as oesophagitis and failure to thrive and when pharmacological treatment is indicated, PPI are on the front row. However, prescriptions are frequently initiated on the presence of non-specific clinical symptoms, although PPI are thought to be uneffective [8, 33]. Consequently, we selected as primary outcome, an objective marker of acid gastric secretion and not clinical symptoms.

The recruitment period was unexpectedly long, as 50% of neonates screened had normal pH monitoring, confirming the non-specificity of clinical symptoms or were already under PPI treatment, merely upon clinical symptoms and were not eligible for the trial. We also encountered difficulties in including neonates over 35 weeks GA, due to short hospitals stays. Finally, mean *prior* probabilities of success were attributed by neonatal specialists to reflect the potential dose-response relationship (the higher the dose, the higher the response) of omeprazole. Although not totally representative, this method has been used in previous dose-finding trials in neonates using the same Bayesian method [11–13, 34].

## Conclusion

Omeprazole is extensively prescribed in neonates although prescriptions on clinical symptoms are questionable. When treatment is required, the daily dose needs to be validated in preterm and term neonates. We then conducted the first dose finding study for omeprazole in neonates. We used a Bayesian sequential method, and showed that the minimum effective doses differ among neonates. When studied at 35 weeks post-menstrual age or more, premature neonates of less than 32 weeks required a dose of 2.5 mg/kg/day whereas less premature and term neonates required 1 mg/kg/day. Additional studies are required to appraise the differential impact of gestational and postnatal ages to determine dose requirements, taking into account the potential impact of medical conditions and concomitant medications.

## Supporting Information

S1 File Protocol OMEPRAZOLE-1—Recherche de dose et Pharmacocinétique de population de l’Oméprazole chez le nouveau-né en traitement du reflux gastrooesophagien. (PDF)

S1 Table Sequential estimation of *posterior* probabilities of success after each cohort of three patients for the group of neonates born between 32 and 35 weeks of gestational age. Bold characters indicate the estimations of the minimum effective dose after the inclusion of each cohort. Data from the 12 neonates that participated in the first-stage analysis to determine the MED are presented in this table.
(DOC)

S2 Table Sequential estimation of *posterior* probabilities of success after each cohort of three patients for the group of neonates born after 35 weeks of gestational age. At the end of the recruitment, it was not possible to determine the minimum effective dose in the >35 weeks GA study group due to low inclusion rate. Thus, we decided to pool the data with the previous GA study group (32–35 weeks) and analyse the data following the meta-analysis proposed by Zohar and al (2011). The summary of the analysis is given in the table below. The number of observed failures was gathered at each dose level and the observed probabilities of efficacy were estimated. Because of the small number of participants, several dose levels were not used. At the next step, weights for all available doses are provided by a simulation study based on the model of interest and marginal frequencies provided by the observations. The weights were the resulted percentage of the total allocation for each dose level. At the end of the meta-analysis, the pooled recommended dose was of 1mg/kg.
(DOCX)

S3 Table Pooled estimation of the minimum effective dose in the group of infants born after 32 weeks of gestational age. (DOCX)

S1 Fig Mean posterior probability of success related to the minimum effective dose and its 95% credibility interval according to patients’ inclusions in the group of neonates born between 32 and 35 weeks of gestational age (n = 18). CI95%: 95% credibililty interval, MED: Minimum Efficient Dose.
(DOCX)
