# Supplementary material for: Dose-Finding Study of Omeprazole on Gastric pH in Neonates with Gastro-Esophageal Acid Reflux Using a Bayesian Sequential Approach
Source: PLoS One. 2016 Dec 21;11(12):e0166207. doi: 10.1371/journal.pone.0166207 (PMC5176365; doi:10.1371/journal.pone.0166207)
Supplement: S1 File — (PDF) [file pone.0166207.s001.pdf]

PROMOTEUR
Assistance Publique – Hôpitaux de paris
Département de la Recherche et du Développement
Hôpital Saint Louis
1, avenue Claude Vellefaux
75010 - PARIS

PHRC National 2005

OMEPRAZOLE-1 - **Recherche de dose et Pharmacocinétique de population de l'Oméprazole chez le nouveau-né en traitement du reflux gastro-oesophagien**

Professeur Evelyne JACQZ-AIGRAIN
Hôpital Robert Debré- 48 bld Sérurier - 75019 Paris
CIC Pédiatrique 9202 - Unité de Pharmacologie Pédiatrique

Page de SIGNATURE DU PROTOCOLE

Recherche biomédicale N°*P 051043*

Titre : « OMEPRAZOLE 1 - Recherche de dose et Pharmacocinétique de population de l'Oméprazole chez le nouveau-né en traitement du reflux gastro-oesophagien »

Version N°1 du 29/11/ 2006

L'investigateur coordonnateur :

Pr Evelyne Jacqz Aigrain

CIC Pédiatrique 9202 – Unité de Pharmacologie Pédiatrique,

Hôpital Robert Debré
48, boulevard Sérurier
75935 PARIS CEDEX 19

L'investigateur coordonnateur autorise également la diffusion de son identité et de ses coordonnées dans le répertoire des recherches biomédicales

Date : ……………/………/………..

Signature :

Le promoteur :

Dr Olivier CHASSANY

Assistance publique – hôpitaux de Paris
Délégation Interégionale à la Recherche Clinique
Hôpital Saint Louis
75010 PARIS

Date : ……………/………/………..

Signature :

NB : cette version correspond au texte du protocole et annexes adressés au CPP et à l'autorité compétente respectivement pour avis et demande d'autorisation.
Si ensuite une autre version est rédigée suite à des modifications, il faut refaire le circuit des signatures afin d'être toujours à jour des versions du protocole actif.

Plan du dossier

I. RESUME 5
II. ETAT DES CONNAISSANCES SCIENTIFIQUES : OBJECTIFS ET HYPOTHESES DU PROJET 8
1. Le reflux gastro-oesophagien chez le nouveau-né 8
2. Stratégies de prise en charge du reflux gastro-oesophagien et justification de l'essai......9
3. Pharmacologie de l'oméprazole..9
4. Les arguments pour la conduite d'un protocole chez le nouveau-né..............................11
III. PROTOCOLE DE RECHERCHE : Détermination de la dose efficace d'oméprazole 12
1 Introduction 12
2. Objectifs 12
3. Plan expérimental 13
3.1 Choix du plan expérimental..13
3.2 Calcul du nombre de sujets nécessaires..14
3.3 Critères d'inclusion et de non inclusion des patients..16
3.3.1 Critères d'inclusion..16
3.3.2 Critères de non inclusion 17
3.4 Durée de participation à la recherche 17
3.5 Durée totale de la recherche 17
4. Déroulement pratique de l'étude..18
5. ....Administration des traitements 20
5.1 Traitement non médicamenteux du reflux 20
5.2. Traitements médicamenteux par oméprazole 20
Présentation du médicament et AMM..20
Procédures d'attribution de la dose d'oméprazole..21
Modalités de préparation des gélules et de délivrance..21
5.3. Liste des médicaments concomitants autorisés..22
6. Conditions de réalisation de la pHmétrie 22
Réalisation de la pHmétrie..22
Critères d'interprétation de la pHmétrie 23
7. Etudes pharmacocinétique et pharmacogénétique de l'oméprazole 24
7.1 Prélèvements pour l'étude pharmacocinétique 24
7.2 Prélèvement pour l'étude pharmacogénétique. 25
8. Critères de jugement..25
8.1. Critère principal de jugement 25
8.2. Critères secondaires de jugement..26
9. Gestion des données et statistiques 26
Recueil des données cliniques, modalités d'administration des traitements. 26
Rôle du CIC 26
Sorties d'étude..26
10. Analyse des résultats..27
10.1 Recherche de dose 27
10.2 Tolérance 28
10.4 Analyse pharmacocinétique de population..28
11 Comités de l'étude 29
12 Paramètres d'évaluation de la sécurité 30

Déclaration des évènements indésirables graves aux Autorités de Santé....32
Modalités et durée du suivi des personnes suite à la survenue d'évènements indésirables 32
13. Droit d'accès aux données et documents source 33
14. Contrôle et assurance de la qualité 33
Procédures de monitoring 33
Transcription des données dans le cahier d'observation....34
15. Considérations légales et éthiques 34
Demande d'autorisation auprès de l'Afssaps 35
Demande d'avis au Comité de Protection des Personnes....35
Déclaration CNIL 35
Note d'information et Consentement éclairé 36
Rapport final de la recherche 36
16. Traitement des données et conservation des documents et des données relatives à la recherche 36
17. Financement et assurance, si ces points ne font pas l'objet d'un document distinct (par exemple, contrat ou convention). 37
Assurance 37
Engagement scientifique 37
18. Règles relatives à la publication 37

IV : ANNEXES 40
Annexe 1 : liste des centres investigateurs et Services Participant au projet : 40
Annexe 2 : Dossier médicament 42
Annexe 3 : Fiche de déclaration des évènements indésirables graves 46
Annexe 4 : Références bibliographiques 48

I. RESUME

« OMEPRAZOLE 1 - Recherche de dose et Pharmacocinétique de population de l'Oméprazole chez le nouveau-né en traitement du reflux gastro-oesophagien. »

Professeur Evelyne JACQZ-AIGRAIN CIC Pédiatrique 9202 - Unité de Pharmacologie Pédiatrique, Hôpital Robert Debré

Tous les nouveau-nés, incluant les prématurés à partir de 24 semaines d'âge gestationnel, maintiennent un pH gastrique acide dès les premiers jours de vie. Le profil de maturation, en fonction de l'âge gestationnel, de la pompe à protons ATPase dépendante et/ou du système d'activation de cette pompe par l'intermédiaire du calcium intracellulaire n'est pas connu. Il est nécessaire d'évaluer l'efficacité des inhibiteurs de la pompe à protons en fonction de l'âge gestationnel.

L'oméprazole est un médicament inhibiteur de la pompe à protons (IPP) utilisé chez le nouveau-né à terme et prématuré présentant un reflux gastro-oesophagien, compliqué ou non d'oesophagite (ou oesogastrite). Les données pharmacocinétiques, d'efficacité et de tolérance de ce médicament ne sont pas connues dans cette tranche d'âge et le médicament est donc utilisé hors Autorisation de Mise sur le Marché, dans une pathologie particulière à la période néonatale.

L'Objectif principal de cet essai est d'évaluer la dose minimale efficace d'oméprazole permettant d'obtenir chez le nouveau-né souffrant d'un reflux gastro-oesophagien documenté par pHmétrie, une efficacité à court terme (à 72 heures +ou- 24heures) sur les données de pHmétrie de contrôle.

Les objectifs secondaires sont d'évaluer aux doses utilisées l'effet de l'oméprazole sur le pH oro-pharyngé avant et sous traitement, de réaliser une étude pharmacocinétique de population et pharmacogénétique de l'oméprazole et d'évaluer la tolérance de l'oméprazole dans cette population.

Il s'agit d'une étude en double insu conduite selon une méthode séquentielle bayesienne. Le principe de cette méthode est de déterminer la posologie associée au niveau le plus proche d'un niveau d'efficacité donné (fixé à 95 % dans cette étude), dans une gamme de 5 doses différentes. Cette recherche sera stratifiée sur l'âge de l'enfant en 3 groupes : âge gestationnel (AG) < 32 semaines, AG de 32-35 semaines et AG≥36 semaines.

Les critères d'inclusion sont les suivants : nouveaux nés à terme ou prématurés présentant une pHmétrie pathologique, réalisée hors étude, (temps de pH < 4 supérieur ou égal à 5 % du temps d'enregistrement), recevant une alimentation discontinue par biberons, consentement signé par les deux parents, affiliés à un régime de sécurité sociale ou bénéficiant de la CMU et hospitalisés dans le service de néonatologie de l'Hôpital Robert Debré.

Les critères de non inclusion sont les suivants : Nouveau-né ayant reçu de l'oméprazole ou d'autres inhibiteurs de la pompe à proton avant l'inclusion et notamment avant l'arrivée en néonatologie, Nouveau-né présentant des troubles digestifs (diarrhée principalement), Nouveau-né présentant des perturbations du bilan biologique réalisé pour sa prise en charge, avant la participation au protocole, comme des perturbations hématologiques : leucopénie ou thrombopénie inférieures à deux fois la valeur normale pour l'âge ou des transaminases supérieures à deux fois la normale pour l'age

Le protocole est interrompu après la réalisation de la seconde pHmétrie réalisée 72 ± 24 heures après la première administration d'oméprazole. Cette pHmétrie est réalisée pour la prise en charge habituelle des patients présentant un reflux gastro-oesophagien et non pour le protocole. Le traitement par oméprazole du protocole est alors interrompu et le traitement du RGO poursuivi selon les modalités habituelles du service. La durée du traitement par oméprazole dans les conditions de l'essai est de 96 heures au maximum.

Pour chaque patient, la période de participation est de 96 heures avant la pHmétrie plus 24 heures correspondant à l'enregistrement du résultat de la pHmétrie, (± 48 heures de sécurité) soit une période de participation de 168 heures maximum.

Des prélèvements sanguins supplémentaires seront effectués pour les études pharmacocinétique et pharmacogénétique :

- Un prélèvement de 0,5 ml pour les enfants d'âge gestationnel inférieur à 32 semaines et 2 prélèvements de 0,5 ml pour les autres enfants pour l'étude pharmacocinétique.

- Un prélèvement de 3 ml de sang sera réalisé avec le bilan de sortie pour étudier les caractéristiques génétiques de l'élimination de l'oméprazole.

Le critère de jugement est un critère binaire de type succès ou échec.

Le succès est défini par la normalisation de la pHmétrie à 72 ± 24 heures (après le début du traitement, définie à un temps de pH acide < 4 pendant moins de 5 % du temps d'enregistrement).

L'échec est défini par un temps de pH acide < 4 pendant 5 % ou plus du temps d'enregistrement lors de la pHmétrie réalisée à 72 ± 24 heures après le début du traitement.

Les critères secondaires de jugement sont les modifications des autres paramètres de pHmétrie (Nombre de reflux moyen par heure, Durée du reflux le plus long (définissant la clairance oesophagienne), les modifications du taux de pH mesuré dans la salive en utilisant un papier pH, l'amélioration des symptômes cliniques de RGO à 72 heures et 120 heures, l'amélioration du résultat de la fibroscopie lorsqu'elle est réalisée et l'évaluation de la tolérance en recherchant les effets secondaires possibles

II. ETAT DES CONNAISSANCES SCIENTIFIQUES : OBJECTIFS ET HYPOTHESES DU PROJET

1. LE REFLUX GASTRO-OESOPHAGIEN CHEZ LE NOUVEAU-NE

Tous les nouveau-nés, incluant les prématurés à partir de 24 semaines d'âge gestationnel, maintiennent un pH gastrique acide dès les premiers jours de vie. Par contre, la quantité d'acide formée est moins importante chez le prématuré que chez le nouveau-né à terme (Boyle 2003).

Le reflux gastro-oesophagien (RGO) du nouveau-né à terme et prématuré est une pathologie fréquente (Ewer 2005).

Les manifestations cliniques immédiates du RGO sont diverses (Coletti 2003):

1) au plan clinique, ce sont des rejets, vomissements, bradycardies, désaturations, malaises. La difficulté du diagnostic clinique est liée au fait que les symptômes peuvent relever d'une autre étiologie telles que apnées du prématuré et/ou infection.

2) au plan fibroscopique, oesophagite et/ou gastrite et/ou duodénite.

Le RGO peut être alimentaire et/ou acide. Le RGO acide est objectivé par la pHmétrie oesophagienne qui apprécie le temps de pH < 4 pendant la durée de l'enregistrement, en règle sur 24 heures. Un temps de reflux acide < 4 pendant 5% ou plus du temps d'enregistrement est reconnu comme pathologique chez le nourrisson.

Les chiffres d'incidence sont donc fonction des modalités du diagnostic : clinique exclusif, pHmétrique et/ou fibroscopique. Cette dernière investigation n'évalue que les complications du reflux.

L'incidence du RGO significatif sur le plan clinique est estimé à 22.5% chez le nouveau-né prématuré d'âge gestationnel inférieur à 34 semaines. Lorsque est réalisée une pHmétrie, l'incidence est estimée à 40 à 60 % chez le prématuré et de 20 à 30 % chez le nouveau-né à terme (Ewer 2005, Colletti 2003).

2. STRATEGIES DE PRISE EN CHARGE DU REFLUX GASTRO-OESOPHAGIEN ET JUSTIFICATION DE L'ESSAI

Les stratégies de prise en charge du RGO en période néonatale sont très variables d'une équipe à l'autre et d'un pays à l'autre, en l'absence de tout essai clinique évaluant l'efficacité des diverses stratégies utilisées.

Le traitement non médicamenteux du RGO repose sur l'épaississement des biberons, la position surélevée sur le dos ou le côté.

Le traitement médicamenteux du RGO est plus fréquemment prescrit en cas de reflux compliqué en association au traitement non médicamenteux.

Il peut s'agir :

1) soit d'un prokinétique (Primpéran* ou Motilium* en fonction de l'habitude des prescripteurs) ou en l'absence d'efficacité, de l'urécholine parfois utilisée à la place du prokinétique habituel.

2) soit d'un traitement antihistaminique H2 (cimétidine ou ranitidine)

3) soit un médicament inhibiteur de la pompe à protons au premier rang desquels **l'oméprazole**.

Ce dernier est facile à administrer aux nouveau-nés car il est présenté sous forme de micro granules gastro-protégées. Ces micro granules peuvent être conditionnées en pharmacie dans des gélules à doses adaptées au nouveau-né puis ouvertes et administrées dans un petit volume d'eau. Cependant, les indications, la posologie et la durée d'utilisation de l'oméprazole ne font l'objet d'aucun consensus pendant les premières semaines de vie, en l'absence de toute évaluation de son efficacité et de sa tolérance chez le nouveau-né à terme ou prématuré.

Ce traitement médicamenteux est habituellement administré durant l'hospitalisation et puis jusqu'à la consultation du premier mois ou il est le plus souvent interrompu, en l'absence de signes cliniques inquiétants (persistance de la symptomatologie, prise de poids insuffisante).

3. PHARMACOLOGIE DE L'OMEPRAZOLE

L'oméprazole est un médicament inhibiteur de la pompe à protons située dans les cellules pariétales de la muqueuse gastrique. Ce médicament est largement utilisé

aussi bien chez l'adulte que chez l'enfant.

Il a été largement évalué chez le sujet adulte volontaire sain et malade, et chez l'enfant dans le cadre de pathologies liées à l'hyperacidité gastrique. (Walt 1985 ; Lind 1986 ; Cederberg 1992 ; Faure 2001).

L'oméprazole est détruit en milieu acide et est donc administré sous forme gastro-résistante.

Son absorption a lieu au niveau du grêle. Sa liaison aux protéines plasmatiques est élevée, environ 95%. Le médicament est éliminé par biotransformations hépatiques cytochrome P450 dépendantes. Le cytochrome P450 CYP2C19 est le principal cytochrome impliqué dans le métabolisme de l'oméprazole. Il présente un polymorphisme pharmacogénétique, 3 à 5% des sujets adultes étant métaboliseurs lents. Le métabolisme de l'oméprazole en hydroxyoméprazole est alors faible et la demi-vie du médicament est allongée. Sa demi-vie sérique chez l'adulte est d'environ 40 minutes chez les métaboliseurs rapides.

L'inhibition de la sécrétion acide gastrique est liée à l'aire sous la courbe des concentrations plasmatiques en fonction du temps. Elle est ainsi plus importante chez les métaboliseurs lents.

Cette molécule est disponible sous forme orale et intraveineuse. Cette molécule a une AMM validée chez l'adulte et l'enfant de plus de 1 an.

Malgré l'intérêt potentiel de cette molécule en période néonatale, il n'existe aucune donnée disponible chez le nouveau-né, ni pharmacocinétiques, ni pharmacodynamiques (Kearns, 2003, 1-2). De plus, le profil de maturation de l'activité CYP2C19 n'est pas connu. Les données in vitro (microsomes hépatiques humains) suggèrent cependant une activité métabolique faible à la naissance, chez le nouveau-né.

Les particularités pharmacologiques chez le nouveau-né sont possibles ;

1) le profil de maturation, en fonction de l'age gestationnel, de la pompe à protons ATPase dépendante et/ou du système d'activation de cette pompe par l'intermédiaire du calcium intracellulaire n'est pas connu.

2) les biotransformations de ce médicament sont partiellement immatures chez le nouveau-né, d'autant plus qu'il est prématuré.

4. LES ARGUMENTS POUR LA CONDUITE D'UN PROTOCOLE CHEZ LE NOUVEAU-NE

Les arguments sont nombreux :

1. Indication thérapeutique potentielle dans la prise en charge à court et plus long terme du reflux gastro-oesophagien. Les données d'efficacité sont essentielles dans cette tranche d'âge, car la pathologie est à l'évidence différente de celle de l'adulte et ses complications potentiellement graves.
2. Immaturité et variabilité métabolique et pharmacocinétique (liée aux facteurs génétiques et au développement) sont très importantes chez le nouveau-né. Les études pharmacocinétiques de population peuvent permettre d'identifier les facteurs de variabilités pharmacocinétiques avec un nombre réduit de prélèvements par enfant.
3. Les données de tolérance sont indispensables, en raison des différences de maturation et des risques des médicaments sur un organisme en développement, c'est-à-dire jusqu'à la fin de la puberté.

L'absence d'Autorisation de Mise sur le Marché pour le nouveau-né est liée à l'absence de protocole d'évaluation de l'efficacité et de la tolérance de ce médicament dans cette tranche d'âge. Il s'agit d'une décision de l'industriel reposant sur divers arguments :

1. Difficultés de mise en place d'un essai en néonatalogie
2. Marché du médicament relativement réduit
3. Galénique complexe
4. Médicament déjà disponible en générique

A l'inverse, ce médicament est largement utilisé par les néonatalogistes, non seulement en France mais de manière plus large en Europe et aux Etats-Unis en raison de son efficacité (impressions cliniques lors de thérapeutiques individuelles) et de sa bonne tolérance (avec absence de tout effet indésirable rapporté dans la littérature ou observé en clinique hospitalière).

III. PROTOCOLE DE RECHERCHE : Détermination de la dose efficace d'oméprazole

1 INTRODUCTION

Tous les nouveau-nés, incluant les prématurés à partir de 24 semaines d'âge gestationnel, maintiennent un pH gastrique acide dès les premiers jours de vie. Par contre, la quantité d'acide formée est moins importante chez le prématuré que chez le nouveau-né à terme.

Le profil de maturation, en fonction de l'âge gestationnel, de la pompe à protons ATPase dépendante et/ou du système d'activation de cette pompe par l'intermédiaire du calcium intracellulaire n'est pas connu.

Ceci explique qu'il soit nécessaire d'évaluer l'efficacité des inhibiteurs de la pompe à protons en fonction de l'âge gestationnel.

L'oméprazole est un médicament inhibiteur de la pompe à protons (IPP) utilisé chez le nouveau-né à terme et prématuré présentant un reflux gastro-oesophagien, compliqué ou non d'oesophagite (ou oesogastrite). Les données pharmacocinétiques, d'efficacité et de tolérance de ce médicament ne sont pas connues dans cette tranche d'âge et le médicament est donc utilisé hors Autorisation de Mise sur le Marché, dans une pathologie particulière à la période néonatale.

Le protocole présenté ici a pour but d'évaluer la place de ce médicament dans la prise en charge thérapeutique du reflux gastro-oesophagien chez le nouveau-né à terme et prématuré et hospitalisé en néonatalogie.

Il sera suivi d'un autre protocole évaluant l'efficacité de l'oméprazole à court terme sur les bradycardies néonatales par un essai clinique randomisé oméprazole versus placebo.

2. OBJECTIFS

<u>Objectif principal</u>

Evaluer la dose minimale efficace d'oméprazole permettant d'obtenir chez le nouveau-né souffrant d'un reflux gastro-oesophagien documenté par pHmétrie, une efficacité à court terme (72 + ou - 24 heures) sur les données de pHmétrie de

contrôle.

Objectifs secondaires

Evaluer la tolérance de l'oméprazole chez les nouveaux nés.

Evaluer aux doses utilisées l'effet de l'oméprazole sur les symptômes cliniques dans les 120 heures suivant l'initiation du traitement..

Evaluer aux doses utilisées l'effet de l'oméprazole sur le pH oro-pharyngée en comparant les pH oro-pharyngés avant et sous traitement.

Etudier la corrélation entre la mesure du pH oropharyngé et les résultats de la pHmétrie

Réaliser une étude pharmacocinétique de population et pharmacogénétique de l'oméprazole.

3. PLAN EXPERIMENTAL

3.1 Choix du plan expérimental

Il s'agit d'une étude en double insu conduite selon la méthode de réévaluation séquentielle basée sur une approche bayesienne. Le principe de cette méthode est de déterminer la posologie associée au niveau le plus proche d'un niveau d'efficacité donné (fixé à 95 % dans cette étude), dans une gamme de 5 doses différentes. Le critère de jugement est binaire (succès/échec). Cette recherche sera stratifiée sur l'âge de l'enfant en 3 groupes : âge gestationnel (AG) < 32 semaines, AG de 32-35 semaines et AG≥36 semaines. Ces trois groupes d'enfants seront gérés indépendamment.

Définitions :

L'âge gestationnel s'exprime en semaines d'aménorrhée révolues à partir du premier jour des dernières règles normales. Il mesure la durée entre le premier jour des dernières règles normales et la naissance.

L'âge post natal est l'âge de l'enfant à partir de la date de naissance.

L'âge post conceptionnel est l' âge gestationnel plus l'âge post natal.

Dans chacun des 3 groupes d'âge gestationnel, chaque cohorte de 3 enfants (inclusion groupée) est incluse après que le résultat des 3 enfants de la cohorte

précédente a été enregistré. La dose administrée à chaque cohorte de 3 enfants est celle dont la probabilité de succès calculée *a posteriori*, est la plus proche du niveau d'efficacité recherché de 95 %. Ce type d'étude nécessite un effectif maximal de 30 enfants par groupe de stratification, soit 90 enfants au total. On utilisera une approche en 2 étapes:

1. Recherche de la dose minimale efficace (DME) parmi les 5 doses du panel (voir E1.10).
2. Estimation de la probabilité de réponse associée à la DME.

3.2 Calcul du nombre de sujets nécessaires

Les caractéristiques de la méthode séquentielle bayesienne ont démontré que l'inclusion de 30 patients par groupe d'âge est suffisante pour ce type d'étude. Du fait de différences de maturation en particulier pharmacologique liés à l'âge gestationnel (affectant les différentes étapes de la disposition du médicament et en particulier les activités enzymatiques de métabolisme et la cible du médicament), cette recherche de dose se fera dans 3 groupes d'âge (AG<32, AG 32-35, AG≥36) et donc 90 enfants au maximum seront inclus. Des règles d'arrêt seront appliquées séquentiellement pour éventuellement arrêter les inclusions avant cet effectif (O'Quigley 1990, Zohar, Chevret 2003).

Méthode de réévaluation séquentielle *(MRS)*

5 doses sont disponibles pour l'étude, chacune étant associée à une probabilité de succès *a priori* déterminée par les investigateurs avant le début de l'étude, au vu des informations disponibles sur la relation effet dose. Ces estimations initiales sont les suivantes :

**Dose en mg/kg**	**Probabilité de succès *a priori***
1	50%
1,5	70%
2	85%
2,5	95%

3	100%

Chez le jeune enfant la posologie est de 10 mg/jour. La dose maximale pour un nouveau né sera 15 mg/jour.

L'objectif de l'étude est de déterminer la dose associée à une probabilité de succès de 95 %. Les enfants sont inclus séquentiellement et une probabilité de succès est calculée *a posteriori* pour chaque dose et dans chaque groupe de stratification, en appliquant le théorème de Bayes.

Etape 1 : L'expérimentation commence au signalement d'une inclusion par l'investigateur au biostatisticien, une dose arbitraire est choisie (parmi les 5) par le biostatisticien (pour maintenir l'insu). Cette dose est communiquée au pharmacien qui prépare le traitement pour l'enfant mais l'investigateur est en insu de la dose reçue par l'enfant. La réponse du premier trio d'enfants à cette dose sera communiquée par l'investigateur à ce même biostatisticien qui déterminera la dose suivante à choisir, correspondant à celle dont l'estimation de la probabilité de succès *a posteriori* est la plus proche de 95 %. Le calcul de la probabilité de succès pour chacune des 5 doses est donc calculée *a posteriori*. Cette analyse sera réalisée séquentiellement après l'observation de 3 réponses dans un même groupe d'âge (inclusion groupée avec cohorte de taille 3 à la même dose).

Le double insu est maintenu pendant l'administration du produit et pendant toute la durée de cette étape.

1. Cette étape se termine soit au terme de l'inclusion de 30 enfants pour le groupe d'âge considéré (effectif reconnu comme suffisant dans la littérature sur la MRS [O'Quigley et al, 1990; Chevret, 1993],
2. soit du fait d'une décision d'arrêt liée à l'application des 2 critères suivants:
   - Soit il y a haute probabilité que la dose administrée reste inchangée pour les 2 prochaines cohortes, *P(dose inchangée|data, z=6)>0.85*, alors arrêt de la première étape et passage en deuxième étape
   - Soit arrêt pour l'inefficacité, i.e, aucune dose n'est assez

efficace par rapport au pré requis de l'étude, $P(d_5 < cible) > 0.85$, alors arrêt de l'étude.

La décision d'arrêt de cette première étape ou de l'étude pour chaque strate sera prise par l'ensemble des coordinateurs et statisticiens de l'étude après consultation du comité de pilotage.

Etape 2 : Le restant des patients à inclure de la strate est inclus à la dose retenue à la fin de la première étape pour un groupe d'âge donné. Cette étape se termine soit (1) après l'inclusion du nombre maximal d'enfants prévus dans la strate, soit (2) si il y a une faible probabilité que l'addition de 2 cohortes (hypothétiques) supplémentaires apportera peu sur la précision de la probabilité de succès associée à la dose retenue, P(max de la largeur de l'intervalle de crédibilité |data, z=6)<0.05.

Dans ce cas la décision d'arrêt de l'étude pour chaque strate sera prise par l'ensemble des coordinateurs et statisticiens de l'étude après consultation du comité de pilotage.

3.3 Critères d'inclusion et de non inclusion des patients

3.3.1 Critères d'inclusion

1 Nouveaux nés à terme ou prématurés ayant atteint au moins l'âge post-conceptionnel de 35 semaines présentant une pH métrie pathologique (temps de pH < 4 supérieur ou égal à 5 % du temps d'enregistrement)

Indication de pHmétrie :

- Soit systématique : il s'agit des nouveau-nés nés à un terme inférieur à 30 SA et ayant atteint l'âge post conceptionnel de 35 semaines
- Soit présentant des signes cliniques évocateurs de reflux gastro-oesophagien (malaise, signes respiratoires…) et ayant atteint l'âge post conceptionnel de 35 semaines

2 Recevant une alimentation discontinue par biberons

3 Consentement écrit des deux parents (des titulaires de l'autorité parentale)

4 Affilié à un régime de sécurité sociale ou bénéficiant de la CMU

5 Hospitalisés dans le service de néonatologie de l'Hôpital Robert Debré.

3.3.2 Critères de non inclusion

1 Nouveau-né ayant reçu de l'oméprazole ou un autre inhibiteur de la pompe à proton avant l'inclusion et notamment avant l'arrivée en néonatologie

2 Nouveau-né présentant des troubles digestifs (diarrhée principalement)

3 Nouveau-né présentant des perturbations du bilan biologique réalisé pour sa prise en charge :

- perturbations hématologiques : leucopénie ou thrombopénie inférieures à deux fois la valeur normale pour l'âge
- transaminases supérieures à deux fois la normale pour lâge

4 Absence de consentement des titulaires de l'autorité parentale

3.4 Durée de participation à la recherche

Le protocole est interrompu après la réalisation de la seconde pHmétrie réalisée 72 ± 24 heures (Compte tenu des rendez-vous de pHmétrie) après la première administration d'oméprazole, la durée de traitement avant la réalisation de la pHmétrie étant de 48 heures au minimum et de 96 heures au maximum. Cette pHmétrie est réalisée pour la prise en charge habituelle des patients présentant un reflux gastro-oesophagien et non pour le protocole. Le traitement de l'enfant par oméprazole dans le cadre de cet essai est alors interrompu et le traitement du RGO poursuivi selon les modalités habituelles du service.

Pour chaque patient, la période de participation dure un maximum de 96 heures avant la pHmétrie plus 24 heures correspondant à l'enregistrement de la pHmétrie, soit une durée de participation de 168 heures au maximum (pour avoir 48 heures de marge).

La période d'exclusion est de 7 jours.

3.5 Durée totale de la recherche

Selon les données des services de néonatalogie et pour l'année 2004 :

Le diagnostic de reflux gastro-oesophagien a été porté sur des données pHmétriques en 2004 chez 100 nouveau-nés dans le service à l'hôpital Robert Debré.

Par conséquent, nous estimons à 18 mois la durée nécessaire pour inclure 90 patients.

4. DEROULEMENT PRATIQUE DE L'ETUDE

a Le reflux gastro-oesophagien est diagnostiqué sur les résultats de la pHmétrie réalisée dans un but thérapeutique (dans le cadre des soins) après l'autonomie alimentaire (c.à.d enfant alimenté par biberons)

b une pHmétrie pathologique est définie par une durée de pH < 4 supérieure ou égale à 5 % du temps d'enregistrement.

c La prise en charge non médicamenteuse du reflux gastro-oesophagien est systématique

d L'enfant bénéficie durant tout le protocole du traitement non médicamenteux du reflux gastro-oesophagien qui inclut

- Le couchage en excluant le décubitus et le procubitus ventral
- L'épaississement du lait selon les modalités habituelles du service : soit lait pré-épaissi soit épaississement par Magicmix , Gélopectose ou Gumilk.

e L'enfant est inclus après consentement des deux parents, une fois le résultat de la pHmétrie obtenu par un médecin senior du service de néonatalogie, vérifiant l'existence du reflux gastro-oesophagien.

f Attribution des traitements : La dose à administrer au premier enfant inclus dans l'essai sera choisie par le biostatisticien parmi les 5 doses à tester (voir paragraphe 3.1). La posologie à administrer au premier enfant inclus dans l'essai sera communiquée au pharmacien par le biostatisticien (entre 1 et 3 mg/kg/j). L'heure de la première administration d'oméprazole sera notée dans le cahier d'observation et la durée du traitement sera de 48 à 96 heures

g Dans le délai nécessaire à l'obtention du traitement (gélules adaptées au poids de l'enfant), la détermination du pH oro-pharyngé sera réalisée en utilisant un simple papier pH à 2 reprises à 3 heures d'intervalle et juste avant la prise d'un biberon

Après la première administration est réalisé un prélèvement sanguin veineux pour l'étude pharmacocinétique de population. Un prélèvement (0.5 ml) est réalisé chez les nouveaux-nés de moins de 32 semaines d'âge gestationnel et 2 chez ceux d'age gestationnel supérieur ou égal à 32 semaines.

Le prélèvement est effectué soit entre H0.5 et H4 soit entre H4 et H12, soit les deux (ces temps de prélèvements étant définis à partir des données pharmacocinétiques disponibles chez le grand enfant et chez l'adulte, et correspondant respectivement à un prélèvement précoce durant des phases d'absorption - distribution et un prélèvement durant la phase d'élimination). Le prélèvement est combiné dans la mesure du possible à un bilan biologique afin de réduire le nombre de prélèvements sanguins. Ce prélèvement est réalisé après application de crème EMLA. Pour les enfants de moins de 32 semaines d'âge gestationnel, le prélèvement est réalisé à l'un des deux temps pré-définis, et contemporain d'un bilan sanguin dans le cadre des soins.

h La pHmétrie est contrôlée au bout de 72 ± 24 heures après le début du traitement (48 heures au minimum et 96 heures au maximum).

i La détermination du pH oro-pharyngé sera réalisée en utilisant un simple papier pH à 2 reprises juste avant et juste après la pose de la sonde de pHmétrie.

Après évaluation du critère de jugement principal, le médecin investigateur communiquera au biostatisticien le numéro de l'enfant inclus (n°patient), et la réponse au traitement (succès/échec). ***Le statisticien, après avoir analysé les résultats de la cohorte de trois patients et calculé les probabilités de succès a posteriori, a connaissance de la dose à attribuer aux 3 enfants suivants. Le statisticien communiquera au pharmacien la posologie (en mg/kg) à administrer à chaque enfant au fur et à mesure des inclusions.***

Lors du bilan de sortie de l'enfant, est réalisé un tube de sang veineux (3 ml sur EDTA) pour l'étude du polymorphisme pharmacogénétique CYP2C19 après signature du consentement pour une étude génétique par les responsables de l'autorité parentale.

5. ADMINISTRATION DES TRAITEMENTS

5.1 Traitement non médicamenteux du reflux

Les patients bénéficient durant le protocole du traitement non médicamenteux du reflux gastro-oesophagien qui inclut :

1. Le couchage en excluant le décubitus et le procubitus ventral
2. L'épaississement du lait selon les modalités habituelles du service : soit lait pré-épaissi soit épaississement par Magicmix , Gélopectose ou Gumilk.

5.2. Traitements médicamenteux par oméprazole

Présentation du médicament et AMM

L'oméprazole est autorisé chez l'enfant. La présentation Mopral® - gélule 10 mg - a une autorisation de mise sur le marché (AMM 559.639.7) pour l'enfant à partir de l'âge de 1 an dans l'indication suivante : oesophagite érosive ou ulcérative symptomatique par reflux gastro-oesophagien.

La posologie est de 10 mg par jour. Les gélules ne pouvant être avalées en dessous de l'age de 5 ans, les gélules contenant des microgranules gastro-protégées peuvent être ouvertes et mélangées à un aliment tel que l'eau, le jus d'orange ou un yaourt.

Pour cette étude, l'oméprazole sera conditionné sous forme de gélules. L'administration de l'oméprazole se fera avec la prise d'un biberon de lait ou d'un biberon d'eau. Les gélules préparées par la pharmacie seront ouvertes et les granules seront introduites dans la bouche du nourrisson et avalées avec la prise d'un biberon.

L'oméprazole n'a pas d'autorisation de mise sur le marché pour le nouveau-né à terme et prématuré. Cependant les doses utilisées varient de 1 à 3 mg/kg /jour en une seule prise, en fonction des prescripteurs. La dose maximale pour les nouveaux nés sera de 15mg/jour.

Choix de la gamme de doses testée dans le protocole

Pour le protocole, la gamme de doses est de 1 à 3 mg/kg en une prise. Elle a été choisie en fonction des données disponibles chez l'enfant plus grand (à partir de 1 an) et de l'expérience des néonatalogistes.

L'oméprazole possède une AMM à la dose de 1mg/kg/jour chez l'enfant au dessus de l'âge de 1 an. Cette posologie a été sélectionnée comme posologie journalière minimale en l'absence d'argument pour une efficacité plus importante chez le nouveau-né.

A l'inverse, la posologie maximale est de 3 mg/kg/jour en raison des observations cliniques, mettant en évidence une absence d'efficacité, chez le nouveau-né, des posologies habituellement utilisées chez l'enfant plus grand (1 à 2 mg/kg/jour), obligeant à envisager une relation dose/efficacité différente chez le nouveau-né

Procédures d'attribution de la dose d'oméprazole

Après vérification des critères d'inclusion et de non inclusion et obtention du consentement informé écrit, le nouveau-né se voit attribuer un numéro de patient (à reporter sur le CRF).

L'attribution de la dose est réalisé par l'Unité d'Epidémiologie Clinique de l'hôpital Robert Debré :

Unité d'Epidémiologie Clinique, Hôpital Robert Debré
Téléphone 01 40 03 23 45 de 9H à 18H
Fax 01 40 03 24 85

Modalités de préparation des gélules et de délivrance

Le traitement par oméprazole est délivré par le pharmacien gérant la préparation et la dispensation du traitement. Des gélules d'oméprazole seront préparées par la

pharmacie de l'hôpital Robert Debré à partir du produit base présenté en granules. Les gélules seront titrées avec précision au dixième de mg (0.1 mg) en fonction du poids de l'enfant.

Le traitement médicamenteux par l'oméprazole sera dans tous les cas administré en association au traitement non médicamenteux habituel : épaississement des biberons, positionnement.
L'ordonnance commandant la gélule est faxée par l'investigateur à la pharmacie. La posologie (en mg/kg) à administrer est faxée par le biostatisticien. Cinq gélules sont préparées en pharmacie pour chaque enfant : quatre gélules pour administration au patient (1 gélule par jour pendant 4 jours maximum), et la cinquième gélule pour vérifier le cas échéant la préparation en dosant son contenu.

5.3. Liste des médicaments concomitants autorisés

Aucun autre traitement médical du RGO n'est autorisé pendant la durée de participation à l'étude.

6. CONDITIONS DE REALISATION DE LA PHMETRIE

Réalisation de la pHmétrie

En période néonatale la pHmétrie est réalisée chez des nouveau-nés recevant une alimentation discontinue par biberons

- Soit à titre systématique chez les nouveau-nés, nés à un terme inférieur à 30 semaines d'aménorrhée (SA) et ayant atteint l'âge post conceptionnel de 35 semaines
- Soit devant des signes cliniques évocateurs de reflux gastro-oesophagien (malaise, signes respiratoires…) chez les nouveau-nés nés à un terme supérieur ou égal à 30 SA et ayant atteint l'âge post conceptionnelde 35 semaines

En raison de leur prématurité, les nouveau-nés nés à un âge gestationnel variant de 25 à 35 semaines sont alimentés par gavage, ce qui impose la mise en place d'une sonde gastrique et rend impossible la réalisation d'une pHmétrie. Lorsqu'ils ont

atteint l'age post-conceptionnel de 35 semaines, ils sont alimentés par biberons et la pHmétrie peut être réalisée.

La pHmétrie est réalisée à partir de l'âge de 35 semaines d'âge post-conceptionnel.

La sonde de pHmétrie doit être placée dans le bas œsophage. La difficulté de mise en place tient à la taille de l'enfant et au fait qu'il bouge beaucoup.
La position de la sonde est calculée en utilisant

1 Soit la formule suivante : (Taille / 4) + 3, qui donne en cm la longueur de la sonde introduite à partir de la bouche
2 Soit par une radiographie contrôlant la position de la sonde deux vertèbres au dessus du diaphragme

Ces deux méthodes peuvent être utilisées dans le présent protocole pour mettre en place la sonde de pHmétrie. Le choix sera laissé au médecin mettant en place la pHmétrie.

L'enregistrement du pH du bas œsophage est continu sur 24 heures et avec lecture informatique.

Critères d'interprétation de la pHmétrie

Les normes de la pHmétrie chez le nouveau-né à terme ou prématuré restent discutées en raison de la variabilité des conditions de mesures, de la difficulté de réunir des données sur un nombre suffisant d'enfants. Elles sont donc définies à partir des données obtenues chez l'enfant plus grand.

Les critères d'interprétation de la pHmétrie sont basés sur les paramètres suivants :

- Index de reflux : temps global de reflux défini par un pH inférieur à 4 (pendant les 24 heures d'enregistrement)- Un reflux pathologique est défini par un temps de pH inférieur à 4 pendant 5% ou plus des 24 heures d'enregistrement.

Sont aussi utilisés les paramètres suivants :

- Nombre de reflux moyen par heure
- Durée du reflux le plus long (définissant la clairance oesophagienne)

En dessous de 35 semaines, la mesure du pH oro-pharyngé est une technique non invasive pour établir le diagnostic de reflux gastro-oesophagien acide. Les données sont encore limitées mais sur 23 nouveau-nés prématurés ayant eu une pHmétrie, la mesure du pH oro-pharyngé avait une sensibilité de 89%, une spécificité de 80%, une valeur prédictive positive de 94% et une valeur prédictive négative de 67% (James, Ewer 1999°). L'intérêt de ce test doit donc être validé sur de plus grandes séries et sera réalisé dans le présent protocole.

7. ETUDES PHARMACOCINETIQUE ET PHARMACOGENETIQUE DE L'OMEPRAZOLE

7.1 Prélèvements pour l'étude pharmacocinétique

Les prélèvements sanguins ont pour objectifs de déterminer les paramètres pharmacocinétiques de population et leurs facteurs de variabilité liés à l'âge. Les enfants d'age gestationnel inférieur à 32 semaines ont 1 prélèvement (0.5ml), les autres 2.

Au maximum, quatre vingt dix enfants sont prévus et 150 prélèvements maximum seront donc disponibles pour l'étude de pharmacocinétique de population. Ces prélèvements seront utilisés pour déterminer les concentrations plasmatiques de l'oméprazole et de son métabolite l'hydroxyoméprazole. Ils permettront en particulier d'étudier l'impact de l'âge gestationnel et l'âge post-natal sur la pharmacocinétique de l'oméprazole chez le nouveau-né.

Gestion des Biothèques

Les prélèvements pour dosage de l'oméprazole et de son métabolite sont centrifugés pour séparer le plasma. Celui-ci est conservé à -20°C au laboratoire de pharmacologie. Les biothèques seront conservée**s dans l'**Unité de pharmacologie de l'hôpital Robert Debré et dans l'Unité de biochimie génétique de l'hôpital Robert Debré sous la responsabilité du Pr Evelyne Jacqz-Aigrain et du Pr Jacques Elion respectivement. Ces échantillons seront conservés jusqu'à la publication des résultats de l'étude.

Dosages de l'oméprazole et de l'hydroxyoméprazole

Ces dosages seront réalisés dans le laboratoire de Pharmacologie Pédiatrie, par technique d'HPLC/UV en utilisant des techniques mises au point dans le laboratoire.

Les techniciens réalisant ces dosages sont Michel Popon et Marie Hélène Quernin.

7.2 Prélèvement pour l'étude pharmacogénétique.

Un prélèvement de 3 ml sur EDTA est réalisé au moment du bilan de sortie de l'enfant qui permet l'extraction de DNA et l'étude du polymorphisme cytochrome P450 2C19 impliqué dans la variabilité du métabolisme de l'oméprazole.

Gestion des prélèvements.

Les prélèvements pharmacogénétiques sont enregistrés et stockés au CRB de l'hôpital Robert Debré (Professeur Jacques Elion) selon les procédures habituelles jusqu'à la réalisation des techniques de biologie moléculaire permettant l'étude du polymorphisme CYP2C19. Durée de conservation : Après réalisation des examens et analyse des résultats, au maximum trois ans après la fin de l'étude.
Il n'y aura pas d'utilisation des prélèvements pour une autre étude.

Génotypage de CYP2C19

Le génotype CYP2C19 sera réalisé par méthode Taqman selon une méthode disponible au laboratoire de Pharmacologie Pédiatrique et Pharmacogénétique sous la responsabilité de Yves Médard, technicien de laboratoire.

8. CRITERES DE JUGEMENT

8.1. Critère principal de jugement

Le critère de jugement est un critère binaire de type succès ou échec.

1 Le succès est défini par la normalisation de la pHmétrie à 72 $\pm$ 24 heures (entre 48 et 96 heures) après le début du traitement, définie par un temps de pH acide (< 4) strictement inférieur à 5 % du temps d'enregistrement)

2 L'échec est défini par un temps de pH acide (< 4) supérieur ou égal à 5 % du temps d'enregistrement lors de la pHmétrie réalisée à 72H (entre 48 et 96 heures) après le début du traitement

8.2. Critères secondaires de jugement

a. Modification des autres paramètres de pHmétrie Nombre de reflux moyen par heure
b. Durée du reflux le plus long (définissant la clairance oesophagienne)

1 Variation du pH mesuré dans la salive en utilisant un papier pH entre l'inclusion et la fin de l'essai
2 Amélioration des symptômes cliniques de RGO à 72 heures et 120 heures
3 Amélioration de la fibroscopie lorsqu'elle est réalisée

Evaluation de la tolérance en recherchant les effets secondaires possibles

9. GESTION DES DONNEES ET STATISTIQUES

Recueil des données cliniques, modalités d'administration des traitements.

Un cahier de recueil des données, transmis à l'équipe de néonatologie collaborant à cette étude est rempli pour chaque malade. Chaque cahier porte le numéro du patient dans l'étude, le numéro du traitement reçu par le patient, et les initiales du patient. Il comprend aussi les antécédents maternels et de la grossesse, l'histoire néonatale et les symptomes cliniques du RGO, les principales constantes biologiques, les modalités d'administration du médicament étudié , l'évolution du RGO et résultats de la pHmétrie .

La survenue des effets indésirables est notée pour tous les malades.

Rôle du CIC

Les inclusions seront suivies mensuellement par l'attaché de recherche clinique de l'URC en charge du protocole.

La coordination du protocole est assurée par le TEC du CIC de l'investigateur coordonnateur.

Sorties d'étude

Une sortie d'étude peut être réalisée à la demande des parents ou pour tout effet indésirable grave.

10. ANALYSE DES RESULTATS

L'analyse statistique sera réalisée sous la responsabilité du Dr Corinne Alberti, Unité d'Epidémiologie Clinique, hôpital Robert Debré, mise à part la partie pharmacocinétique de population qui sera réalisé par Caroline Monchaud

10.1 Recherche de dose

On utilisera une approche bayesienne de conduite et d'estimation de la DME. Cette analyse sera réalisée séquentiellement après l'observation des réponses d'une cohorte de trois enfants d'un même groupe d'âge (inclusion groupée avec cohorte de taille 3 à la même dose).

Cette étape se termine soit au terme de l'inclusion de 30 enfants pour le groupe d'age considéré (effectif reconnu comme suffisant dans la littérature sur la MRS [O'Quigley et al, 1990; Chevret, 1993], soit du fait d'une décision d'arrêt liée à l'application des 2 critères suivants:

- Soit il y a haute probabilité que la dose administrée reste inchangée pour les 2 prochaines cohortes, *P(dose inchangée|data, z=6)>0.85*, alors arrêt de la première étape et passage en deuxième étape.
- Soit arrêt pour l'inefficacité, i.e, aucune dose n'est assez efficace par rapport au pré requis de l'étude, $P(d_5<cible)>0.85$, alors arrêt de l'étude.

La décision d'arrêt de cette première étape ou de l'étude pour chaque strate sera prise par l'ensemble des coordinateurs et statisticiens de l'étude après consultation du comité de pilotage.

2ème étape : Le restant des patients à inclure de la strate est inclus à la dose retenue à la fin de la première étape. Cette étape se termine soit (1) après l'inclusion du nombre maximal d'enfants prévus dans la strate, soit (2) si il y a une faible probabilité que l'addition de 2 cohortes (hypothétiques) supplémentaires apportera peu sur la précision de la probabilité de succès associée à la dose retenue, P(max de la largeur de l'intervalle de crédibilité |data, z=6)<0.05.

Dans ce cas la décision d'arrêt de l'étude pour chaque strate sera prise par

l'ensemble des coordinateurs et statisticiens de l'étude après consultation du comité de pilotage.

10.2 Tolérance

Dans chaque groupe de dose sera estimée la fréquence des effets indésirables.

La tolérance de l'oméprazole est très bonne chez l'enfant et l'adulte. Les effets indésirables mentionnés ci-dessous sont rares.

Les effets indésirables suivants seront recherchés systématiquement et colligés dans le cahier d'observation.

- Troubles du transit à type de diarrhée ou constipation
- Numération formule sanguine : neutropénie, thrombopénie
- Elévation des transaminases

L'évaluation des effets indésirables cliniques sera réalisée tous les jours. L' administration étant de courte durée, l'évaluation de la tolérance biologique sera basée sur les bilans biologiques précédant l'inclusion et suivant la fin du protocole, utilisant les bilans réalisés dans le cadre des soins, les plus proches de ces deux événements.

Tous les autres effets indésirables seront notés dans le CRF.

10.3 Analyse des objectifs secondaires

Un test de Wilcoxon pour séries appariées sera utilisé pour tester entre l'inclusion et la fin de l'essai la variation du pH mesuré dans la salive.

Le nombre de reflux moyen par heure et la durée du reflux le plus long seront comparés entre les doses par des tests de Kruskall Wallis.

Le coefficient de corrélation de Spearman servira à étudier le lien entre la mesure du pH oro-pharyngée et les résultats de la pHmétrie.

10.4 Analyse pharmacocinétique de population

Le protocole permettra de déterminer les concentrations plasmatiques de l'oméprazole obtenues après la première administration par voie orale. Les paramètres pharmacocinétiques de population et leur variabilité, en fonction de la

dose, de l'âge gestationnel post-natal, du poids seront déterminés en utilisant le logiciel NONMEM.
Les effets d'autres facteurs seront analysés : Apgar, état pathologiques (retard de croissance intra-utérin, infection maternofoetale, etc...).

NONMEM est une méthode paramétrique d'estimation des caractéristiques de population en pharmacocinétique. Cette méthode ajuste simultanément les données obtenues chez tous les individus inclus dans l'étude, pour une molécule donnée, et estime les paramètres pharmacocinétiques moyens, leur variance liée aux variations interindividuelles et la variance résiduelle.
L'effet de covariances discrètes ou continues sur les paramètres pharmacocinétiques peut être secondairement testé par l'intermédiaire de modèles statistiques dit de second niveau, basées sur des équations mathématiques liant les paramètres et les covariances (le plus souvent des relations linéaires).
Cette méthode sera utilisée pour l'analyse des données de concentrations de l'oméprazole avec un modèle spécifique d'absorption pour la forme orale.

L'étude pharmacocinétique de population permettra de définir les paramètres pharmacocinétiques de l'oméprazole dans la population étudiée et de quantifier l'impact de l'âge gestationnel et post-natal sur les paramètres définis : clairance de l'oméprazole, volume de distribution, demi-vie d'élimination.
D'autres facteurs de variabilité de recherches seront liés aux paramètres biologiques (protéinémie, hématocrite).
Les résultats permettront d'établir des recommandations posologiques en fonction de l'âge.

11 COMITES DE L'ETUDE

Comité de pilotage :

Il sera constitué des initiateurs cliniciens du projet, du biostatisticien en charge du projet, des représentants du promoteur AP-HP à la DRCD et dans l'URC nommés pour cette recherche.

Il définira l'organisation générale et le déroulement de la recherche et coordonnera les informations.

Il déterminera initialement la méthodologie et décidera en cours de recherche des conduites à tenir dans les cas imprévus, surveillera le déroulement de la recherche en particulier sur le plan de la tolérance et des évènements indésirables.

Evelyne Jacqz Aigrain, Corinne Alberti, Christophe Aucan.

Comité indépendant de surveillance des évènements indésirables :

Il a une fonction consultative et décisionnelle lorsque le promoteur fait appel à lui sur des points médicaux tels la tolérance et les événements indésirables. Il est constitué de personnes extérieures à la recherche dont nécessairement un clinicien spécialiste de la pathologie étudiée et un pharmacologue/pharmacovigilant et selon le protocole un méthodologiste/biostatisticien.

Il a pour Rôle et compétence :

Valider les évènements critiques déclarés par les investigateurs.

Poursuite/Arrêt d'étude

Liste des Evènements cliniques à déclarer : Tous les EIG SONT à déclarer, aucun autre effet indésirables n'est attendu.

Les membres seront nommés par le promoteur.

12. PARAMETRES D'EVALUATION DE LA SECURITE

- **Evènement indésirable**

  Toute manifestation nocive survenant chez une personne qui se prête à une recherche biomédicale que cette manifestation soit liée ou non à la recherche ou au produit sur lequel porte cette recherche.

- **Effet indésirable d'un médicament expérimental**

  Toute réaction nocive et non désirée à un médicament expérimental quelle que soit la dose administrée

- **Evènement ou effet indésirable grave**

  Tout évènement ou effet indésirable qui entraîne la mort, met en danger la vie de la personne qui se prête à la recherche, nécessite une hospitalisation ou la prolongation de l'hospitalisation, provoque une incapacité ou un handicap importants ou durables, ou bien se traduit par une anomalie ou une

malformation congénitale, et s'agissant du médicament, quelle que soit la dose administrée.

- **Effet indésirable inattendu d'un médicament expérimental**

Tout effet indésirable dont la nature, la sévérité ou l'évolution ne concorde pas avec les informations figurant dans le résumé des caractéristiques du produit lorsque le médicament est autorisé, et dans la brochure pour l'investigateur lorsqu'il n'est pas autorisé.

- **Fait nouveau**

Toute nouvelle donnée de sécurité, pouvant conduire à une réévaluation du rapport des bénéfices et des risques de la recherche ou du médicament expérimental, ou qui pourrait être suffisant pour envisager des modifications dans l'administration du médicament expérimental, dans la conduite de la recherche.

Procédures mises en place en vue de l'enregistrement et de la notification des évènements indésirables :

Evènements indésirables non graves :
Tout événement indésirable - non grave suivant la définition précédente - observé lors de la recherche et dans ses suites devra être reporté dans le cahier d'observation dans la section prévue à cet effet.
Un seul évènement doit être reporté par item. L'évènement peut correspondre à un symptôme, un diagnostic ou à un résultat d'examen complémentaire jugé significatif. Tous les éléments cliniques ou para-cliniques permettant de décrire au mieux l'évènement correspondant doivent être reportés.

Evènements indésirables graves (EIG) :
Les investigateurs doivent notifier **immédiatement** au promoteur AP-HP les évènements indésirables graves tels que définis ci-dessus.
L'investigateur complète les formulaires d'évènements indésirables graves (du cahier d'observation de la recherche) et les envoie au DRCD par fax au 01 44 84 17 99 et ce, dans les 48 heures (après si possible un appel téléphonique immédiat au 01 44 84 17 23 en cas de décès ou d'une menace vitale inattendus).

L'investigateur doit également informer l'URC en charge de la recherche de la survenue de l'EIG.

Pour chaque évènement indésirable grave, **l'investigateur devra émettre un avis sur le lien de causalité de l'évènement avec chaque médicament expérimental et les autres traitements éventuels.**

L'obtention d'informations relatives à la description et l'évaluation d'un évènement indésirable peuvent ne pas être possibles dans le temps imparti pour la déclaration initiale.

Aussi, l'évolution clinique ainsi que les résultats des éventuels bilans cliniques et des examens diagnostiques et/ou de laboratoire, ou toute autre information permettant une analyse adéquate du lien de causalité seront rapportés :

- **soit sur la déclaration initiale d'EIG s'ils sont immédiatement disponibles,**
- **soit ultérieurement et le plus rapidement possible, en envoyant par fax une nouvelle déclaration d'EIG complétée (et en précisant qu'il s'agit d'un suivi d'EIG déclaré et le numéro de suivi).**

Toutes les déclarations faites par les investigateurs devront identifier chaque sujet participant à la recherche **par un numéro de code unique** attribué à chacun d'entre eux.

En cas de décès notifié d'un sujet participant à la recherche**, l'investigateur communiquera au promoteur tous les renseignements complémentaires demandés** (compte-rendu d'hospitalisation, résultats d'autopsie…).

Tout fait nouveau survenu dans la recherche ou dans le contexte de la recherche, provenant de données de la littérature ou de recherches en cours, devra être notifié au promoteur.

Déclaration des évènements indésirables graves aux Autorités de Santé

Elle sera assurée par le Pôle de Pharmacovigilance du DRCD, après évaluation de la gravité de l'évènement indésirable, du lien de causalité avec chaque médicament expérimental et les autres traitements éventuels ainsi que du caractère inattendu des effets indésirables.
Toutes les suspicions d'effet indésirable grave inattendu seront déclarées par le promoteur aux autorités compétentes dans les délais légaux.
En cas d'effet indésirable grave inattendu dû à l'un des traitements de la recherche ou à la recherche elle-même, les autorités compétentes, le Comité de Protection des Personnes et les investigateurs de la recherche devront être informés.

MODALITES ET DUREE DU SUIVI DES PERSONNES SUITE A LA SURVENUE D'EVENEMENTS INDESIRABLES

Tout patient présentant un évènement indésirable doit être suivi jusqu'à la résolution ou la stabilisation de celui-ci.

- Si l'évènement n'est pas grave, l'évolution en sera notée sur la page correspondante du cahier d'observation à la section prévue à cet effet.

- Si l'évènement est grave, un suivi d'EIG sera envoyé au DRCD.

13. DROIT D'ACCES AUX DONNEES ET DOCUMENTS SOURCE

Les personnes ayant un accès direct conformément aux dispositions législatives et réglementaires en vigueur, notamment les articles L.1121-3 et R.5121-13 du code de la santé publique (par exemple, les investigateurs, les personnes chargées du contrôle de qualité, les moniteurs, les assistants de recherche clinique, les auditeurs et toutes personnes appelées à collaborer aux essais) prennent toutes les précautions nécessaires en vue d'assurer la confidentialité des informations relatives aux médicaments expérimentaux, aux essais, aux personnes qui s'y prêtent et notamment en ce qui concerne leur identité ainsi qu'aux résultats obtenus. Les données collectées par ces personnes au cours des contrôles de qualité ou des audits.

14. CONTROLE ET ASSURANCE DE LA QUALITE

La recherche sera encadrée selon les procédures opératoires standard du promoteur.
Le déroulement de la recherche dans les centres investigateurs et la prise en charge des sujets sera faite conformément à la déclaration d'Helsinki et les Bonnes Pratiques en vigueur.

Procédures de monitoring

Les ARC représentants du promoteur effectueront des visites des centres investigateurs au rythme correspondant au schéma de suivi des patients dans le protocole, aux inclusions dans les différents centres et au niveau de risque D qui a été attribué à la recherche.

Visite d'ouverture de chaque centre : avant inclusion, pour une mise en place du protocole et prise de connaissance avec les différents intervenants de la recherche biomédicale.

Lors des visites suivantes, les cahiers d'observation seront revus au fur et à mesure de l'état d'avancement de la recherche par les ARC. L'investigateur principal de chaque centre ainsi que les autres investigateurs qui incluent ou assurent le suivi des personnes participant à la recherche s'engagent à recevoir les ARC à intervalles réguliers.
Lors de ces visites sur site et en accord avec les Bonnes Pratiques Cliniques, les éléments suivants seront revus :

- Respect du protocole et des procédures définies pour la recherche,
- Vérification des consentements éclairés des patients
- Examen des documents source et confrontation avec les données reportées dans le cahier d'observation quant à l'exactitude, les données manquantes, la cohérence des données selon les règles édictées par les procédures du DRCD.

- Visite de fermeture : bilan à la pharmacie, documents de la recherche biomédicale, archivage.

Transcription des données dans le cahier d'observation

Toutes les informations requises par le protocole doivent être fournies dans le cahier d'observation et une explication donnée par l'investigateur pour chaque donnée manquante.
Les données devront être transférées dans les cahiers d'observation au fur et à mesure qu'elles sont obtenues qu'il s'agisse de données cliniques ou para-cliniques. Les données devront être copiées de façon nette et lisible à l'encre noire dans ces cahiers (ceci afin de faciliter la duplication et la saisie informatique).
Les données erronées dépistées sur les cahiers d'observation seront clairement barrées et les nouvelles données seront copiées sur le cahier avec les initiales et la date par le membre de l'équipe de l'investigateur qui aura fait la correction.
L'anonymat des sujets sera assuré par un numéro de code et les initiales de la personne qui se prête à la recherche sur tous les documents nécessaires à la recherche, ou par effacement par les moyens appropriés des données nominatives sur les copies des documents source, destinés à la documentation de la recherche.

Les données informatisées sur un fichier seront déclarées à la CNIL selon la procédure adaptée au cas.

15. CONSIDERATIONS LEGALES ET ETHIQUES

Le promoteur est défini par la loi 2004-806 du 9 août 2004. Dans cette recherche, l'AP-HP est le promoteur et le Département de la Recherche Clinique et du Développement (DRCD) en assure les missions réglementaires.

Remarques :
Avant de démarrer la recherche, chaque investigateur fournira au représentant du promoteur de la recherche une copie de son ***curriculum vitæ personnel daté et signé*** *et comportant son numéro d'inscription à l'Ordre des Médecins.*

La version du protocole avec ses annexes sera signée conjointement par l'investigateur coordonnateur et le représentant du promoteur avant demande d'autorisation à l'Afssaps et demande d'avis du CPP. Le cas échéant, le responsable scientifique sera également signataire.

Le DRCD doit être informé de tout projet de modification du protocole par l'investigateur coordonnateur.
Les modifications devront être qualifiées en substantielles ou non.
Une modification substantielle est une modification susceptible, d'une manière ou d'une autre, de modifier les garanties apportées aux personnes qui se prêtent à la

recherche biomédicale (modification d'un critère d'inclusion, prolongation d'une durée d'inclusion, participation de nouveaux centres.....).

Toute modification substantielle devra faire l'objet ***par le promoteur après paiement d'une taxe*** *d'une demande d'autorisation auprès de l'Afssaps et/ou d'une demande d'avis du CPP.*

Demande d'autorisation auprès de l'Afssaps

Pour pouvoir démarrer la recherche, l'AP-HP en tant que promoteur doit soumettre un dossier de demande d'autorisation auprès de l'autorité compétente l'Afssaps. L'autorité compétente, définie à l'article L. 1123-12, se prononce au regard de la sécurité des personnes qui se prêtent à une recherche biomédicale, en considérant notamment la sécurité et la qualité des produits utilisés au cours de la recherche conformément, le cas échéant, aux référentiels en vigueur, leur condition d'utilisation et la sécurité des personnes au regard des actes pratiqués et des méthodes utilisées ainsi que les modalités prévues pour le suivi des personnes.

Demande d'avis au Comité de Protection des Personnes

En accord avec l'article L.1123-6 du Code de Santé Publique, le protocole de recherche doit être soumis par le promoteur à un Comité de Protection des Personnes L'avis de ce comité est notifié à l'autorité compétente par le promoteur avant le démarrage de la recherche.

Après le commencement de la recherche, toute modification substantielle de celle-ci à l'initiative du promoteur doit obtenir, préalablement à sa mise en oeuvre, un avis favorable du comité et une autorisation de l'autorité compétente. Dans ce cas, si cela est nécessaire, le comité s'assure qu'un nouveau consentement des personnes participant à la recherche est bien recueilli.

Déclaration CNIL

La loi prévoit que la déclaration du fichier informatisé des données personnelles collectées pour la recherche doit être faite avant le début effectif de la recherche.

Une **méthodologie de référence spécifique au traitement de données personnelles opéré dans le cadre des recherches biomédicales définies par la loi 2004-806 du 9 août 2004** car entrant dans le champ des articles L.1121-1 et suivants du Code de Santé Publique a été établie par la CNIL en janvier 2006.

Cette méthodologie permet une **procédure de déclaration simplifiée** lorsque la nature des données recueillies dans la recherche est compatible avec la liste prévue par la CNIL dans son document de référence.
Lorsque le protocole bénéficie d'un contrôle qualité des données par un ARC représentant le promoteur et qu'il entre dans le champ d'application de la

procédure simplifiée CNIL, le DRCD en qualité de promoteur demandera au responsable du fichier informatique de s'engager par écrit sur le respect de la méthodologie de référence MR06001 simplifiée.

Note d'information et Consentement éclairé

> Le consentement écrit doit être recueilli auprès de toute personne se prêtant à la recherche avant la réalisation de tout acte nécessité par la recherche biomédicale.
>
> Prévoir un type de consentement par population concernée (mineur, majeur, …)

Rapport final de la recherche

Le rapport final de la recherche sera écrit en collaboration par le coordonnateur et le biostatisticien pour cette recherche. Ce rapport sera soumis à chacun des investigateurs pour avis. Une fois qu'un consensus aura été obtenu, la version finale devra être avalisée par la signature de chacun des investigateurs et adressée au promoteur dans les meilleurs délais après la fin effective de la recherche. Un rapport rédigé selon le plan de référence de l'autorité compétente doit être transmis à l'autorité compétente ainsi qu'au CPP dans un délai de un an, après la fin de la recherche, s'entendant comme la dernière visite de suivi du dernier sujet inclus. Ce délai est rapporté à 90 jours en cas d'arrêt prématuré de la recherche.

16. TRAITEMENT DES DONNEES ET CONSERVATION DES DOCUMENTS ET DES DONNEES RELATIVES A LA RECHERCHE

Les documents d'une recherche entrant dans le cadre de la loi sur les recherches biomédicales doivent être archivés par toutes les parties pendant une durée de 15 ans après la fin de la recherche.

Cet archivage indexé comporte :

- Les copies de courrier d'autorisation de l'Afssaps et de l'avis obligatoire du CPP
- Les versions successives du protocole (identifiées par le n° de version et la date de version),
- Les courriers de correspondance avec le promoteur,
- Les consentements signés des sujets sous pli cacheté (dans le cas de sujets mineurs signés par les titulaires de l'autorité parentale) avec la liste ou registre d'inclusion en correspondance,
- Le cahier d'observation complété et validé de chaque sujet inclus,
- Toutes les annexes spécifiques à l'étude,

- Le rapport final de l'étude provenant de l'analyse statistique et du contrôle qualité de l'étude (double transmis au promoteur).
- Les certificats d'audit éventuels réalisés au cours de la recherche

La base de données ayant donné lieu à l'analyse statistique doit aussi faire l'objet d'archivage par le responsable de l'analyse (support papier ou informatique).

17. Financement et assurance, si ces points ne font pas l'objet d'un document distinct (par exemple, contrat ou convention).

Assurance

L'Assistance Publique-Hôpitaux de Paris est le promoteur de cette recherche. En accord avec la loi sur les recherches biomédicales, elle a pris une assurance auprès de la compagnie GERLING KONZERN pour toute la durée de la recherche, garantissant sa propre responsabilité civile ainsi que celle de tout intervenant (médecin ou personnel impliqué dans la réalisation de la recherche) (loi n°2004-806, Art L.1121-10 du CSP).
L'Assistance Publique - Hôpitaux de Paris se réserve le droit d'interrompre la recherche à tout moment pour des raisons médicales ou administratives; dans cette éventualité, une notification sera fournie à l'investigateur.

Engagement scientifique

Chaque investigateur s'engagera à respecter les obligations de la loi et à mener la recherche selon les B.P.C., en respectant les termes de la déclaration d'Helsinki en vigueur. Pour ce faire, un exemplaire de **l'engagement scientifique (document type DRCD)** daté et signé **par chaque investigateur** de chaque service clinique d'un centre participant sera remis au représentant du promoteur.

18. Règles relatives à la publication

L'AP-HP est propriétaire des données et aucune utilisation ou transmission à un tiers ne peut être effectuée sans son accord préalable.
Seront premiers signataires des publications, les personnes ayant réellement participé à l'élaboration du protocole et son déroulement ainsi qu'à la rédaction des résultats.

Par précaution, l'ordre des signataires pourra être défini par avance.

L'Assistance Publique- Hôpitaux de Paris doit être mentionnée comme étant le promoteur de la recherche biomédicale et comme soutien financier le cas échéant. Les termes « Assistance Publique- Hôpitaux de Paris » doivent apparaître dans l'adresse des auteurs.

19. CALENDRIER PREVISIONNEL DE DIFFERENTES ETAPES

Etapes déjà effectuées

Décembre 2004 Mise au point du protocole oméprazole avec les médecins
Mars 2005 de Néonatologie
Avril 2005 Appel d'Offres PHRC
novembreDecembre 2006 Demande de Promotion, soumission au CPP et à l'AFSSAPS
janvier 2007 Début des inclusions dans le protocole
août 2008 Informatisation des données
août à novembre 2008 Dosages et Analyses, Résultats, Rédaction

20. PERSPECTIVES LIEES AU PROTOCOLE : IPP CHEZ LE NOUVEAU-NE PREMATURE

Ce protocole permettra

1. de déterminer la dose d'oméprazole efficace à court terme sur le reflux gastro-oesophagien par les modifications de pHmétrie à 72 heures de traitement en période néonatale
2. de définir les paramètres pharmacocinétiques de l'oméprazole et leurs facteurs de variabilité, en particulier l'age (gestationnel et post-natal) et les facteurs pharmacogénétiques

Autres recherches prévues

1. de déterminer si l'oméprazole administrée pendant une période courte en période néonatale à la dose précédente permet de réduire la fréquence de bradycardies graves (fréquence cardiaque inférieure à 80 battements par minute)
2. L'intérêt de déterminer le pH oro-pharyngé dans le RGO du nouveau-né pourra être étudié sur une population plus large que dans l'étude préliminaire déjà publiée chez les enfants ayant aussi une pHmétrie.

Ce protocole permettra de déterminer la dose d'oméprazole efficace pour la prise en charge du RGO chez les nouveau-nés à terme et prématurés pour lequel il n'y a pas de données disponibles et de fournir les recommandations posologiques adaptées aux différentes tranches d'age gestationnel et post-natal.

IV : ANNEXES

ANNEXE 1 : LISTE DES CENTRES INVESTIGATEURS ET SERVICES PARTICIPANT AU PROJET :

1. Centres investigateurs

Equipe de néonatalogie

- Service de Néonatalogie - Hôpital Robert Debré - Professeur Aujard

Ce service est spécialisé en Néonatalogie et notamment dans la prise en charge du nouveau-né prématuré et son recrutement est important. Il est impliqué dans de nombreux projets de prise en charge des pathologies néonatales et collabore avec le centre d'investigation clinique de l'hôpital.

Liste des investigateurs

Pr Aujard, Dr Belaich, Dr Olivier Baud, Dr Caroline Farnaux

2. Services Participant au projet

CIC pédiatrique

- CIC Hôpital Robert Debré - Professeur Evelyne Jacqz-Aigrain Ce CIC est exclusivement pédiatrique

Service gastroentérologie **:** Docteur Marc Bellaiche - Hôpital Robert Debré - Paris.
Marc Bellaiche est responsable de l'unité d'explorations fonctionnelles digestives dans le service de gastro-entérologie (Professeur Cezard).

Service de Pharmacologie pédiatrique et pharmacogénétique – Hôpital Robert Debré - L'hôpital Robert Debré a la mission en collaboration avec les services cliniques et le CIC d'augmenter les connaissances sur les médicaments pédiatriques utilisés en dehors d'une autorisation de mise sur le marché. Il a été à l'origine ou a participé à plusieurs protocoles d'évaluation des IPP en pédiatrie (oméprazole, lanzoprazole - protocole industriel). Le laboratoire fait aussi partie du CIB Hématologie-Oncologie et greffes de l'hôpital Saint Louis et dans ce cadre met au point les dosages de

médicaments pour la recherche clinique

Le dosage de l'oméprazole et hydroxyoméprazole a été mis au point pour un protocole précédent par chromatographie liquide et détection ultraviolette.

La collaboration est étroite avec le Service de pharmacie (Docteur Rieutord), dans le cadre du centre commun de dosages des médicaments (CPDM) ou sont réalisés les interprétations pharmacocinétiques et en particulier les analyses pharmacocinétiques de population dont l'équipe a l'expérience (Burtin - 1994)

Caroline Monchaud, AHU de pharmacologie.

Service de Pharmacie **-** Hôpital Robert Debré : Professeur Brion - Docteur Olivier Bourdon et Docteur André Rieutord.

Préparation des gélules d'oméprazole à dose adaptée à chaque nouveau-né, en fonction de procédures de standardisation.

Unité d'Epidémiologie Clinique **:** Hôpital Robert Debré - Docteur Corinne Alberti**.**

L'unité d'épidémiologie clinique a un rôle central dans la conduite des différentes étapes de ce protocole, notamment :

La gestion des aspects statistiques : méthode séquentielle bayesienne

La responsabilité des bases de données

La gestion des analyses statistiques

Unité de Recherche Clinique Paris Nord : Hôpital Bichat - Docteur Florence Tubach

Estelle Marcault (ARC)

Gestion et coordination du projet

Mise en place technico-réglementaire et logistique de l'essai

Monitoring

Département de la Recherche Clinique et du Développement

Christophe Aucan et Shohreh Azimi.

Gestion, soutien et coordination, Soumission du protocole, Demande d'autorisation

Gestion financière, Soutien logistique

Centre de Ressources Biologiques Hopital Robert Debré - Professeur Jacques Elion

Mise en baque des prélèvements pharmacogénétiques

ANNEXE 2 : DOSSIER MEDICAMENT

1) Circuit

A l'inclusion, l'investigateur faxera au pharmacien une ordonnance (document 1) où sera préciser l'âge gestationnel, le poids, les 2 premières lettres du nom et la première du prénom, le numéro d'inclusion dans l'étude de l'enfant (inclusion chronologique), et le nom du protocole.

Le pharmacien faxera cette ordonnance au statisticien qui lui indiquera dans la partie réservée à cet effet la dose en mg/kg à attribuer à cet enfant.

Le pharmacien préparera les gélules nécessaires au traitement de l'enfant plus une pour vérification de la dose, soit 5 gélules au totale (4 jours maximum de traitement). Les gélules seront transmises à l'investigateur accompagnée d'une feuille de route (document 2).

2) Préparation des gélules d'oméprazole

Les gélules seront préparées à partir des gélules de MOPRAL® 10 mg en fonction de la dose attribuée par l'unité d'épidémiologie clinique et en fonction du poids de l'enfant à la réception de l'ordonnance.

Technique de fabrication des gélules d'oméprazole :

- **a** Calculer la dose d'oméprazole à administrer au patient :
  Dose = posologie x poids enfant en Kg
- **b** Ouvrir n gélules de Mopral® en fonction de la dose à administrer pour 5 gélules
- **c** Peser la dose à administrer au dixième de mg près (Conserver le ticket de pesée)
- **d** Mettre cette dose dans une gélule
- **e** Répéter les opérations c) et d) pour les 4 autres gélules
- **f** Conditionner 4 gélules sous blister et en garder 1 (avec le ticket de pesée) pour le contrôle de la dose
- **g** Etiqueter le blister au nom de l'étude
- **h** Envoyer ce blister au médecin investigateur avec la feuille de route

Protocole OMEPRAZOLE 1
Pr Jacqz Aigrain

Nom I__I__I Prénom I__I
N°Patient I__I__I

3) Feuille de route et ordonnance

a Document 1

ORDONNANCE

PROTOCOLE OMEPRAZOLE 1

Pr Jacqz Aigrain

Nom l__l__l

Prénom l__l

Poids de l'enfant l__l__l__l__l g

N°patient l__l__l

Age gestationnel : ☐ strictement inférieur à 32 semaines

☐ entre 32 à 35 semaines

☐ supérieur ou égal à 36 semaines

Dr……………………………………………………………Le

Signature

Cadre réservé à l'unité d'épidémiologie clinique :

Dose à attribuée au patient :

☐ 1 mg/kg

☐ 1.5 mg/kg

☐ 2 mg/kg

☐ 2.5 mg/kg

☐ 3 mg/kg

Nom, date et signature :

I__I__I I__I__I I__I__I__I__I

Cadre réservé au pharmacien

Par (Nom et Signature) :

Dose dans chaque gélule : l__l__l , l__l mg

Gélule préparée le l__l__l l__l__l l__l__l__l__l

Numéro de lot ___________________

N° de traitement

N° de fax

Cette ordonnance sera archivée par la pharmacie avec les tickets de pesée de préparation des gélules.

b Document 2

FEUILLE DE ROUTE

PROTOCOLE OMEPRAZOLE 1

Pr Jacqz Aigrain

A l'attention du Dr ______________________________, Service ______________________

Patient : Nom l__l__l

Prénom l__l

N°patient l__l__l

Nombre de gélules remises : l__l

Gélules préparées le l__l__l l__l__l l__l__l__l__l

Par (Nom et Signature) :

N° de lot

N° de traitement.

ANNEXE 3 : FICHE DE DECLARATION DES EVENEMENTS INDESIRABLES GRAVES

FICHE D'OBSERVATION D'UN EVENEMENT INDESIRABLE GRAVE SUSCEPTIBLE D'ETRE DU A UNE RECHERCHE BIOMEDICALE	ASSISTANCE PUBLIQUE HÔPITAUX DE PARIS

Cette fiche doit être retournée dûment complétée recto-verso au DRCD à l'attention de Christophe Aucan ou Shohreh Azimi par fax : 01 44 84 17 99

Date de déclaration : I__I__I I__I__I I__I__I__I__I (jj/mm/aaaa)
Titre de l'étude : OMEPRAZOLE 1 - Recherche de dose et Pharmacocinétique de population de l'Oméprazole chez le nouveau-né en traitement du reflux gastro-oesophagien.(**P051043**)

Centre n° I__I__I__I **Investigateur :** ____________________
Nom et adresse du centre : ____________________

____

Identification du patient

Date d'inclusion I__I__I I__I__I I__I__I__I__I
Nom I__I__I__I Prénom : I__I__I
Patient n° I__I__I__I
Date de naissance I__I__I I__I__I I__I__I__I__I
Age I__I__I__I mois
Poids I__I__I__I kg
Taille I__I__I__I cm
Sexe Masculin ☐ Féminin ☐

Evénement indésirable grave

décès
mise en jeu du pronostic vital
nécessite ou prolonge l'hospitalisation
séquelles durables
anomalie congénitale
autre préciser)____________________

Lévée d'insu ____________________**oui ☐ non ☐**
Si oui préciser traitement ____________________**posologie** __________ **en mg/kg/jour**
N° de traitement : ____________________

Antécédents (allergies, insuffisance rénale….) **:** ____________________

____

____

____

Description de l'événement indésirable :
Date de survenue I__I__I I__I__I I__I__I__I__I et heure de survenue I__I__I I__I__I
jj mm aaaa hh min

____

____

____

Délai de survenue : ____________________
Médicaments associés (à l'exclusion de ceux utilisés pour traiter l'événement) :

nom commercial ou DCI	voie	dose/24h	date début	date fin (si arrêt) sinon en cours	indication

Si dispositif médical associé :
Modèle / type / référence : ________________________________
Nom du fabricant : ____________________ du fournisseur ____________________
N° de série ou de lot :__________________ date de péremption si stérile I__I__I I__I__I I__I__I__I__I
Evolution *(indiquez si des mesures symptomatiques ont été prises) :*

____

____

Date de disparition |__|__| |__|__| |__|__|__|__| et heure de disparition |__|__| |__|__|
jj mm aaaa hh min

Autres étiologies envisagées, examens complémentaires réalisés et résultats :

____

____

Selon l'investigateur, l'événement indésirable semble plutôt lié :

à un problème d'utilisation d'un matériel
à un problème technologique
à un médicament administré
aux procédures de l'étude
à la progression de la maladie
à une maladie intercurrente
autre, préciser : ________________________________

Date : I__I__I I__I__I I__I__I__I__I Nom : **Signature :**

PARTIE RESERVEE AU PROMOTEUR : NE PAS REMPLIR

Numéro d'identification de l'événement :
date de réception par le promoteur I__I__I I__I__I I__I__I__I__I
date de ce rapport : I__I__I I__I__I I__I__I__I__I initial suivi n° ________
Selon le promoteur, l'événement indésirable semble plutôt lié :

☐à un problème technologique (matériel)
☐à un médicament administré
☐aux procédures de l'étude
☐à la progression de la maladie
☐à une maladie intercurrente
☐autre, préciser : ____________________

Si selon le promoteur, l'événement semble plutôt lié au protocole :

☐l'événement indésirable est attendu
☐ l'événement indésirable est inattendu

Commentaires du promoteur :

____

____

Nom et qualité du représentant du promoteur : Le Représentant Légal de la DDRC
Signature :

ANNEXE 4 : RÉFÉRENCES BIBLIOGRAPHIQUES

Beal SL, Sheiner LB (1980) The NONMEM system. Am. Statist. 34:118-119.

B. Blondel, P. Truffert, A. Lamarche-Vadel, M. Dehan, B. Larroque, Le groupe epipage. (2003). Utilisation des services médicaux par les grands prématurés pendant la première année de vie dans la cohorte Epipage. Archives de Pédaitrie. 960-968.

Boyle JT. Acid secretion from birth to adulthood. J Paediatrics (2003) 37:S12-S16.

Burtin P, Mallet A, Jacqz-Aigrain E (1991) Population pharmacokinetics of midazolam in neonates. New strategies in drug development and clinical evaluation : the population approach conference. NONMEM USER GROUP. Manchester

Burtin P, Jacqz-Aigrain E, Girard P, Lenclen R, Magny JF, Betremieux P, Tehiry C, Desplanques L, Mussat P. (1994) Population pharmacokinetics of midazolam in neonates. Clin Pharmacol Ther 56:615-625.

Cederberg C, Thomson AB, Mahachai V, Westin JA, Kirdeikis P, Fisher D, Zuk L, Marriage B. Effect of intravenous and oral omeprazole on 24-hour intragastric acidity in duodenal ulcer patients. Gastroenterology 1992;103:913-8.

Colleti RB, Di Lorenzi. Overview of pediatric gastroesophageal reflux disease and proton pump inhibitor therapy (2003) J Ped Gastroenterol Nutrition 37: S7-S11.

Dodge WF, Jelliffe RW, Richardson CJ, McCleery RA, Hokanson JA, Snodgrass WR (1991) Gentamycin population pharmacokinetic models for low birth weight infants using a new non parametric method. Clin. Pharmacol. Ther. 50:25-31.

Ewer A.K MD (2005). Gastro-oesophageal reflux in preterm infants. Communication aux Journées Nationales de Néonatalogie. Paris.

Fattinger K, Vozeh S, Olafsson, A Vleck J, Wenk M, Follath F (1991) Netilmicin in the neonate: population pharmacokinetic analysis and dosing recommendations. 50:55-65.

Faure C, Michaud L, Shaghaghi EK, Popon M, Turck D, Navarro J, Jacqz-Aigrain E. Intravenous omeprazole in children: pharmacokinetics and effect on 24-hour intragastric pH. J Pediatr Gastroenterol Nutr. 2001;33:144-8.

Grasel TH, Donn SM (1985) Neonatal population pharmacokinetics of phenobarbital from routine clinical data. Dev. Pharmacol. Ther. 8:374-383.

James ME, Ewer AK. Acid oro-pharyngeal secretions can predict gastro-oesophageal reflux in preterm infants. Eur J Pediatr 1999; 158: 371é374

John T. Boyle.(2003) Acid secretion form birth to adulthood. Journal of pediatric gastroenterology and nutrition. 37:S12-S16

Karlsson MO, Thomson AH, McGovern EM, Chow P, Evans TJ, Kelman AW (1991) Regulation pharmacokinetics of rectal theophylline in neonates. Ther. Drug. Monit. 13:195-200

Kearns GL, Winter HS. Proton pump inhibitors in pediatrics: relevant pharmacokinetics
and pharmacodynamics. J Pediatr Gastroenterol Nutr. 2003;37 Sl :S52-9.

Kearns GL, Andersson T, James LP, Gaedigk A, Kraynak RA, Abdel-Rahman SM, Ramabadran K, van den Anker JN Omeprazole disposition in children following single-dose administration. J Clin Pharmacol. 2003;43:840-8.

Lind T, Moore M, Olbe L. Intravenous omeprazole: effect on 24-hour intragastric pH in duodenal ulcer patients. Digestion 1986;34:78-86.

Mallet A, Mentré F, Gilles J, Kelman, Thomson AH, Bryson SM, Whiting B (1988) Handling covariates in population pharmacokinetics, with an application to gentamycin. Biomed. Meas. Infor. Contr. 2:138-146.

Mallet A, Mentré F, Steimer JL, Lokiec F (1988) Non parametric maximum likelihood estimation for population pharmacokinetics, with application to cyclosporine. J. Pharmacokinet. Biopharm. 16:311-327.

McGovern EM, Kelman Whiting B, Kelman A.W, Grevel J (1986) Population pharmacokinetics. Theory and clinical application. Clin Pharmacokinet 11:387-401

Moore ES, Faix RG, Banagale RC, Grasela TH (1989) The population pharmacokinetics of theophylline in neonates and young infants. J. Pharmacokinet Biopharm 17:47-66.

O'Quigley J, Pepe M, and Fisher L, Continual reassessment method: a practical design for phase 1 clinical trials in cancer. Biometrics 1990; 46: 33-48 .

Richard B. Colletti, Carlo Di Lorenzo. (2003). Overview of pediatric gastroesophageal reflux disease ans proton pump inhibitor therapy. Journal of pediatric gastroenterology and nutrition 37:S7-11.

Sheiner LB, Grasela TH (1991) An introduction to mixed effect modelling : concepts, definitions and justification. J. Pharmacokinet. Biopharm. 19:11S-24S.

Sheiner L.B, Benet L.Z (1985) Premarketing observational studies of population pharmacokinetics of new drugs. Clin. Pharmacol. Therapeut. 38:481-487.

Thomson A.H, Way S, Bryson SM, A.W, WHITING B (1988) Population pharmacokinetics of gentamicin in neonates. Dev. Pharmacol. Ther. 11:173-179.

Walt RP, Reynolds JR, Langman MJ, Smart HL, Kitchingman G, Somerville KW, Hawkey CJ. Intravenous omeprazole rapidly raises intragastric pH. Gut 1985;26:902-6

Whitehead, J. and I. Stratton (1983). "Group sequential clinical trials with triangular continuation regions." Biometrics 39(1): 227-36.)

Wiest DB, Pinson JD, GAL PS, Brundage RC, Schall S, Ransom JL, Weaver RL, Purohit D, Brow N (1991) Population pharmacokinetics of intravenous indomethacin in neonates with symptomatic patent ductus arterious. Clin Pharmacol Ther 49:550-557.

Zohar S, Chevret S. “Phase I (or phase II) dose-ranging clinical trials: Proposal of a two-stage Bayesian design” Journal of Biopharmaceutical Statistics, 2003 ; 13 : 87-101
