# Supplementary material for: Dose-Finding Study of Omeprazole on Gastric pH in Neonates with Gastro-Esophageal Acid Reflux Using a Bayesian Sequential Approach
Source: PLoS One. 2016 Dec 21;11(12):e0166207. doi: 10.1371/journal.pone.0166207 (PMC5176365; doi:10.1371/journal.pone.0166207)
Supplement: S1 Table — Bold characters indicate the estimations of the minimum effective dose after the inclusion of each cohort. Data from the 12 neonates that participated in the first-stage analysis to determine the MED are presented in this table. (DOC) [file pone.0166207.s002.doc]

**S1 Table. Sequential estimation of *posterior* probabilities of success after each cohort of three patients for the group of neonates born** **between 32 and 35 weeks of gestational age .**

Bold characters indicate the estimations of the minimum effective dose after the inclusion of each cohort.

Data from the 12 neonates that participated in the first-stage analysis to determine the MED are presented in this table.

|  | | | **Dose (mg/kg)** | | | | |
| --- | --- | --- | --- | --- | --- | --- | --- |
|  |  |  | 1 | 1.5 | 2 | 2.5 | 3 |
|  |  |  | **Mean *prior* probabilities of success** | | | | |
|  |  |  | 0.5 | 0.7 | 0.85 | 0.95 | 0.99 |
| Number of cohort | Dose  **(mg/kg daily)** | **Success** | **Mean *posterior* probabilities of success** | | | | |
| 1 | 2 | 3/3 | **0.931** | 0.985 | 0.997 | 1 | 1 |
| 2 | 1 | 3/3 | **0.984** | 0.998 | 1 | 1 | 1 |
| 3 | 1 | 3/3 | **0.992** | 0.999 | 1 | 1 | 1 |
| 4 | 1 | 3/3 | **0.995** | 0.999 | 1 | 1 | 1 |

Second-stage analysis involved another 6 neonates treated at the MED of 1mg/kg with only 1/6 that failed to respond to omeprazole treatment.
