# Supplementary material for: Dose-Finding Study of Omeprazole on Gastric pH in Neonates with Gastro-Esophageal Acid Reflux Using a Bayesian Sequential Approach
Source: PLoS One. 2016 Dec 21;11(12):e0166207. doi: 10.1371/journal.pone.0166207 (PMC5176365; doi:10.1371/journal.pone.0166207)
Supplement: S2 Table — At the end of the recruitment, it was not possible to determine the minimum effective dose in the >35 weeks GA study group due to low inclusion rate. Thus, we decided to pool the data with the previous GA study group (32–35 weeks) and analyse the data following the meta-analysis proposed by Zohar and al (2011). The summary of the analysis is given in the table below. The number of observed failures was gathered at each dose level and the observed probabilities of efficacy were estimated. Because of the small number of participants, several dose levels were not used. At the next step, weights for all available doses are provided by a simulation study based on the model of interest and marginal frequencies provided by the observations. The weights were the resulted percentage of the total allocation for each dose level. At the end of the meta-analysis, the pooled recommended dose was of 1mg/kg. (DOCX) [file pone.0166207.s003.docx]

**S2 Table. Sequential estimation of *posterior* probabilities of success after each cohort of three patients for the group of neonates born after 35 weeks of gestational age.**

Bold characters indicate the estimations of the minimum effective dose after the inclusion of each cohort.

|  | | | **Dose (mg/kg)** | | | | |
| --- | --- | --- | --- | --- | --- | --- | --- |
|  |  |  | 1 | 1.5 | 2 | 2.5 | 3 |
|  |  |  | **Mean *prior* probabilities of success** | | | | |
|  |  |  | 0.5 | 0.7 | 0.85 | 0.95 | 0.99 |
| Number of cohort | Dose  **(mg/kg daily)** | **Success** | **Mean *posterior* probabilities of success** | | | | |
| 1 | 2 | 3/3 | **0.931** | 985 | 0.997 | 1 | 1 |
| 2 | 1 | 3/3 | **0.984** | 0.998 | 1 | 1 | 1 |

At the end of the recruitment, it was not possible to determine the minimum effective dose in the >35 weeks GA study group due to low inclusion rate. Thus, we decided to pool the data with the previous GA study group (32-35 weeks) and analyse the data following the meta-analysis proposed by Zohar and al (2011). The summary of the analysis is given in the table below. The number of observed failures was gathered at each dose level and the observed probabilities of efficacy were estimated. Because of the small number of participants, several dose levels were not used. At the next step, weights for all available doses are provided by a simulation study based on the model of interest and marginal frequencies provided by the observations. The weights were the resulted percentage of the total allocation for each dose level. At the end of the meta-analysis, the pooled recommended dose was of 1mg/kg.
