# Supplementary material for: Dose-Finding Study of Omeprazole on Gastric pH in Neonates with Gastro-Esophageal Acid Reflux Using a Bayesian Sequential Approach
Source: PLoS One. 2016 Dec 21;11(12):e0166207. doi: 10.1371/journal.pone.0166207 (PMC5176365; doi:10.1371/journal.pone.0166207)
Supplement: S3 Table — (DOCX) [file pone.0166207.s004.docx]

**S3 Table. Pooled estimation of the minimum effective dose in the group of infants born after 32 weeks of gestational age.**

|  | **Dose (mg/kg)** | | | | |
| --- | --- | --- | --- | --- | --- |
|  | 1 | 1.5 | 2 | 2.5 | 3 |
|  | **Mean *prior* probabilities of success** | | | | |
|  | 0.5 | 0.7 | 0.85 | 0.95 | 0.99 |
| Number of successes in the 32-35 GA group | 14/15 | - | 3/3 | - | - |
| Number of successes in the > 35 GA group | 3/3 | - | 3/3 | - | - |
| Number of pooled successes | 17/18 | - | 6/6 | - | - |
| Observed success probabilities | 0.94 |  | 1 |  |  |
| Mean relative frequencies of allocation (weight) | 0.12 | 0.31 | 0.15 | 0.13 | 0.12 |
| Pooled estimated Minimum Efficient Dose | **1 mg/kg** | | | | |
