# Supplementary material for: Dose-Finding Study of Omeprazole on Gastric pH in Neonates with Gastro-Esophageal Acid Reflux Using a Bayesian Sequential Approach
Source: PLoS One. 2016 Dec 21;11(12):e0166207. doi: 10.1371/journal.pone.0166207 (PMC5176365; doi:10.1371/journal.pone.0166207)
Supplement: S1 Fig — CI95%: 95% credibililty interval, MED: Minimum Efficient Dose. (DOCX) [file pone.0166207.s005.docx]

**S1 Fig**. **Mean posterior probability of success related to the minimum effective dose and its 95% credibility interval according to patients’ inclusions in the group of neonates born between 32 and 35 weeks of gestational age (n=18).**

CI95%: 95% credibililty interval

MED: Minimum Efficient Dose
